# Perceived stress, stressors, and coping strategies among nursing students in the Middle East and North Africa: an overview of systematic reviews

**DOI:** 10.1186/s13643-021-01691-9

**Published:** 2021-05-05

**Authors:** Sonia Chaabane, Karima Chaabna, Sapna Bhagat, Amit Abraham, Sathyanarayanan Doraiswamy, Ravinder Mamtani, Sohaila Cheema

**Affiliations:** Institute for Population Health, Weill Cornell Medicine–Qatar, Ar-Rayyan, Qatar

**Keywords:** Nursing students, Stress, Stressors, Coping strategies, Systematic review

## Abstract

**Background:**

In nursing students, high stress levels can lead to burnout, anxiety, and depression. Our objective is to characterize the epidemiology of perceived stress, stressors, and coping strategies among nursing students in the Middle East and North Africa region.

**Methods:**

We conducted an overview of systematic reviews. We systematically searched PubMed, Embase, PsycInfo, and grey literature sources between January 2008 and June 2020 with no language restrictions. We included any systematic review reporting measurable stress-related outcomes including stress prevalence, stressors, and stress coping strategies in nursing students residing in any of the 20 Middle East and North Africa countries. We also included additional primary studies identified through a hand search of the reference lists of relevant primary studies and systematic reviews.

**Results:**

Seven systematic reviews and 42 primary studies with data from Bahrain, Egypt, Iraq, Jordan, Oman, Pakistan, Palestine, Saudi Arabia, and Sudan were identified. Most studies included nursing students undergoing clinical training. The prevalence range of low, moderate, and high perceived stress among nursing students was 0.8–65%, 5.9–84.5%, and 6.7–99.2%, respectively. Differences related to gender, training period, or the type of tool used to measure stress remain unclear given the wide variability in the reported prevalence measures across all stress levels. Common clinical training stressors were assignments, workload, and patient care. Academic training-related stressors included lack of break/leisure time, low grades, exams, and course load. Nursing students utilized problem focused (dealing with the problem), emotion focused (regulating the emotion), and dysfunctional (venting the emotions) stress coping mechanisms to alleviate their stress.

**Conclusions:**

Available data does not allow the exploration of links between stress levels, stressors, and coping strategies. Limited country-specific prevalence data prevents comparability between countries. Reducing the number or intensity of stressors through curriculum revision and improving students’ coping response could contribute to the reduction of stress levels among students. Mentorship, counseling, and an environment conducive to clinical training are essential to minimize perceived stress, enhance learning, and productivity, and prevent burnout among nursing students.

**Supplementary Information:**

The online version contains supplementary material available at 10.1186/s13643-021-01691-9.

## Background

Mental health-related conditions are becoming increasingly prevalent among healthcare professionals worldwide [1]. Professions involving constant close human contact and emotional engagement such as nursing, are vulnerable to stress and burnout, which could manifest even before employment [2–4]. A standard baccalaureate nursing program is a very demanding 4-year college or university education [5, 6]. Nursing students experience stress when curricular demands exceed their resources to deal with these demands [7]. Specifically, the clinical training component is dynamic and challenging and was identified as anxiety-producing situations by students during their initial clinical training period [8].

Psychological stress can impact nursing students’ academic and clinical performance [4] as well as their future work life as these may be associated with harmful substance use [9, 10] and reduced empathy [11]. Stress is also associated with serious mental health disorders [12–14] including depression which is one of the leading causes of disability globally [15]. The prevalence of depression among nursing students in Arab states is reported to be 28% [4], approximately six times higher than the prevalence in the general population [16, 17]. Moreover, nursing is a female-dominated profession [4] and evidence shows that female college students [18–20] are more susceptible to depression than their male counterparts [21].

The Middle East, as with many regions worldwide, has a shortage of professional nurses [22, 23]. Published literature has previously reported that a significant percentage of nursing students leave school before program completion [24, 25] as a consequence of stress [26, 27]. Stress reduction programs have been identified to be one of the most effective interventions to decrease attrition in nursing programs [25]. Stress coping strategies are also important determinants that influence overall mental health and well-being [28]. Additionally, published studies report that emotional and behavioral problems, among high stress exposure groups, such as in nursing students may affect their lifetime risk of mental health disorders [29–32]. Understanding stressors that affect nursing students during their training and what coping strategies are utilized by them to address the various stressors is critical. This will enable nursing schools and educators to evaluate and utilize evidence-based interventions and support programs aimed at minimizing attrition in nursing training programs which in turn can help address the shortage of nurses in the region [33].

Several studies [34–46] and systematic reviews [45–47] have assessed stress levels, stressors, and coping strategies among medical students; however, there is a paucity of research and reviews on the subject for nursing students in the region. Our systematic overview synthesizes evidence from published systematic reviews on perceived stress among nursing students in the Middle East and North Africa (MENA) countries. Specifically, we aim to (1) synthesize prevalence data on various stress levels, (2) identify stressors among nursing students, (3) describe stress coping strategies utilized by nursing students in the region, and (4) provide recommendations for stress management.

## Methods

We conducted a systematic overview of published systematic reviews on stress, stressors, and coping strategies among nursing students in the MENA region. Our systematic overview is part of a series of research and publications aimed to improve the quality of evidence generated in the MENA region by synthesizing available literature on population health issues in the region [48–50]. This overview draws from an a priori protocol registered with the International Prospective Register of Systematic Reviews (PROSPERO registration number CRD42017076736) [51]. The methodology of the present systematic overview was informed by the Cochrane Collaboration handbook [52] and was reported following the Preferred Reporting Items for Systematic Reviews and Meta-Analyses (PRISMA) guidelines (Table S1) [53], and the Preferred Reporting Items for Overviews of Systematic Reviews (PRIO-harms) tool (Table S2).

### Search strategy and literature sources

Two independent reviewers (AA and SC1) systematically searched PubMed, Embase, and PsycInfo for any type of review on stress, stressors, and coping strategies on any country in the MENA region published between January 2008 and June 2020. Search terms related to stress, coping strategies/behaviors, and countries’ names were used. The full-search strategy is available in Supplementary, Panel 1 and was validated by a specialized librarian. Additionally, we searched, up to June 2020, literature sources (including grey literature) potentially relevant to the region with no language restrictions including Google Scholar, OpenGrey, E-Marefa, and ALMANHAL platform. We supplemented our literature search by checking the reference lists of relevant included studies and systematic reviews.

### Inclusion and exclusion criteria

In this review, we include countries in the MENA region where Arabic, English, French, and/or Urdu are the primary official languages and/or the medium of instruction in the colleges/universities [51]. The 20 countries included are Algeria, Bahrain, Djibouti, Egypt, Iraq, Jordan, Kuwait, Lebanon, Libya, Morocco, Oman, Pakistan, Palestine, Qatar, Saudi Arabia, Sudan, Syria, Tunisia, the United Arab Emirates (UAE), and Yemen. We included any systematic review reporting measurable stress-related outcomes including stress prevalence, sources of stress, and stress coping strategies or behaviors in nursing students residing in any of the above countries. To ensure a comprehensive up-to-date synthesis of the available data, we also included additional primary studies that had not been identified by included systematic reviews as recommended by the PRIO-harms for preferred reporting items for overviews of systematic reviews [54].

A systematic review was defined as a literature review that had explicitly used a systematic literature search of at least one electronic database to identify all studies that met pre-defined eligibility criteria along with a study selection process [55]. Reviews not reporting a systematic methodology, such as narrative reviews, were excluded. We included published systematic reviews since 2008—the publication year of the first version of the Cochrane Handbook for Systematic Reviews of Interventions [55].

### Data screening and data extraction

Using Rayyan software, duplicates were removed [56]. Two independent reviewers (AA and SB) conducted a multi-stage screening following a standard process. Three reviewers (AA, SB, SC1) independently extracted the data from the included systematic reviews. Discrepancies in the inclusion of systematic reviews and the extracted data were resolved through discussions with the involvement of a fourth reviewer (KC) and under the supervision of the senior authors (SC2 and RM). Extracted data included characteristics of the included systematic reviews as well as the primary studies. From each included systematic review, the following characteristics were extracted: the geographical coverage, literature search period, data literature sources, name of the MENA country for which data was retrieved, along with the number of included studies, targeted review population, and reported stress-related outcomes. From each included primary study, the following characteristics were collected: study design and sample size, years of data collection, population characteristics (type, age, gender), and stress-related outcomes (definition or level, measurement tool, and/or prevalence measure). Study characteristics and any additional data on a stress-related outcome found in an included primary study but not reported by the systematic review were also extracted. In case of discordance between reported data in the systematic review and the primary study, data from the primary study publication was retained.

### Methodological quality assessment

The methodological quality of the included systematic reviews and primary studies was assessed by two independent reviewers (SB, SC1). The AMSTAR measurement tool [57] was used to perform the quality assessment of the included systematic reviews.

A customized tool was used to assess the quality of the included primary studies to accommodate the specific issues related to the methodology and the assessed outcomes. A quality assessment checklist was based on the Cochrane approach for risk of bias (ROB) assessment [58] using an adapted PICOTS framework [59] to assess the quality of included studies with a focus on bias and precision assessment. Classification of studies as low and high risk of bias was based on three quality domains: the description of the study subjects (age and gender), setting (academic year or clinical training), and the validity of the outcome measurement (the use of a validated tool). The precision assessment was based on two quality domains: the sampling methodology (probability-based versus non-probability-based sampling), and the sample size required to reach a study power of at least 80% (≥ 100 versus < 100). For instance, if probability-based sampling was used in a given study, the study was classified with a low (versus high) risk of bias for that domain. Studies were considered as having high (versus low) precision if the total sample size consisted of at least 100 participants. For a perceived stress prevalence of 50% and a sample size of 100, the 95% confidence interval (CI) is 48–52% [60]—a reasonable 95% CI estimate for perceived stress prevalence measure. Studies with missing information for any of the domains were classified as having an unclear risk of bias for that specific domain.

### Synthesis

The characteristics of the included systematic reviews and primary studies were synthesized narratively. To quantify the stress levels among nursing students in the MENA region, available data on the prevalence of perceived stress was summarized using prevalence ranges according to three stress levels: low, moderate, and high, as defined by the different tools utilized in the studies. Prevalence measure variations according to gender, nature of ongoing training, and the type of measurement tools were explored. A measurement tool was considered validated if a validation record in the specific language was retrievable from published literature.

Reported stressors among nursing students are categorized according to the training period: clinical, academic, and stressors external to training periods. For our review, clinical training stressors are classified into six domains as per the perceived stress scale for stressors [61]. The total number of studies reporting each stressor as a source of stress in the study population is also summarized.

Reported stress coping strategies among nursing students in the MENA countries are categorized according to three mechanisms as per the theory of psychological stress and coping [62]: problem-focused (dealing with the problem), emotion-focused (regulating the emotion), and dysfunctional coping (venting the emotions). We summarize the total number of studies reporting each coping mechanism and each specific stress coping strategy.

Recommendations for stress management are synthesized based on the available evidence into three main categories: nursing students, the nursing institutions, and nursing faculty and educators.

## Results

### Characteristics of the included systematic reviews and primary studies

**Fig. 1:**
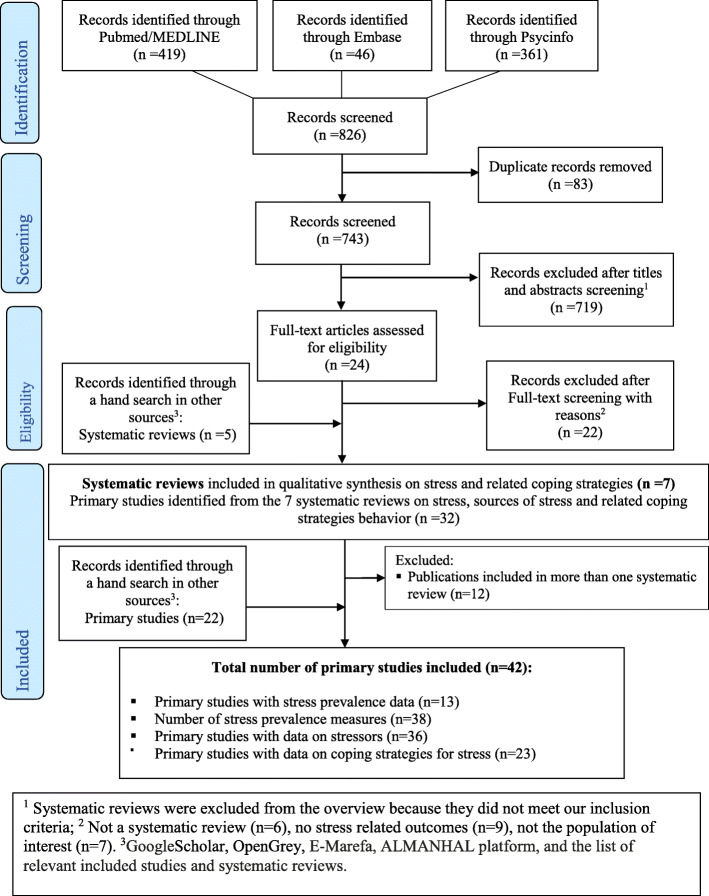
PRISMA 2009 flowchart of the systematic review’s inclusion

In our overview, we include 7 systematic reviews and 42 primary studies on the epidemiology of perceived stress among nursing students containing data for at least 1 MENA country (Fig. 1). The included systematic reviews along with the primary studies are described in Tables S3 and S4, respectively. We found stress-related outcomes for nine MENA countries: Bahrain, Egypt, Iraq, Jordan, Oman, Pakistan, Palestine, Saudi Arabia, and Sudan. The reported primary outcomes are measures of stress levels (six systematic reviews [63–68]) and stress coping strategies (one systematic review [69]). The included systematic reviews did not report stressors as a primary outcome. Five systematic reviews [63–66, 69] searched any country (global coverage), one systematic review [67] searched for data on Saudi Arabia only, and one systematic review [68] searched Asian countries. Thirteen primary studies report prevalence measures on perceived stress, 36 on stressors, and 23 on stress coping strategies.

### Methodological quality assessment of the included systematic reviews and primary studies

Quality assessment results of the systematic reviews included in our overview are described in Table S5. None of the included systematic reviews reported a priori design, the list of excluded studies, or the conflict of interest for the included studies as per the AMSTAR recommendations [57]. Nor did any of the systematic reviews combine primary study findings through meta-analysis. All included systematic reviews conducted a comprehensive literature search and described the characteristics of the included studies. Only two systematic reviews [64, 68] searched grey literature sources. Except for the systematic review of Younas, 2016 [68], all systematic reviews documented the scientific quality of their included studies.

Quality assessment of the included primary studies is summarized in Table S6. All included primary studies provide a detailed description of the study subjects and the research setting. A total of 35 out of 42 primary studies (82.5%) used a validated tool to measure to assess the prevalence of *perceived stress* ((Perceived Stress Scale (PSS), Stress Assessment Scale (SAS), Physio-Psychosocial Stress Scale (PPSS)), *stressors* (PSS, Stressors in Nursing Students Scale (SINS), Student Stress Survey (SSS), Students Stress and Coping Inventory (SSCI), Student Clinical Stressor Scale (SCSS), Student Nurse Stress Index (SNSI)), or *stress coping strategies* ((The Coping Behaviors Inventory (CBI), abbreviated version of the full COPE Inventory (Brief COPE), Coping Orientation to Problems Experienced (COPE), Adolescent Coping Orientation for Problem Experiences (ACOPE), Revised Ways of Coping Strategies Questionnaire (RWCSQ), and Students Stress and Coping Inventory (SSCI)). Only 28 out of 42 primary studies (67.5%) had a sample size of 100 or above and 20 out of 42 primary studies (47.6%) used a probability-based sampling.

### Overview of studies with stress prevalence data

Table 1 summarizes stress prevalence data retrieved from 13 included studies with data from Egypt, Jordan, Iraq, and Saudi Arabia. A total of 38 prevalence measures involving 2804 nursing students were found. Prevalence measures were categorized into three stress levels, low, moderate, or high, as per the tool utilized in the study. Retrieved prevalence data on perceived stress were collected between 2008 and 2019. Most of the included studies involved combined populations of male and female nursing students. Prevalence ranges reported in female only studies were comparable to reported prevalence ranges among combined populations of male and female nursing students. The prevalence range of low perceived stress among all nursing students was 0.8–65%, for moderate perceived stress was 5.9–84.5% and for high perceived stress was 6.7–99.2%. The stress prevalence range among students during the clinical training was comparable to that found in all academic years combined. Twelve out of 13 primary studies with prevalence data utilized a validated tool to measure the prevalence of perceived stress. The PSS was the most widely used psychological instrument for measuring stress perception. We found wide variability in the perceived stress prevalence measures for all stress levels.

**Table 1:** Summary of studies with stress prevalence data among nursing students

**Groups**	**Year of data collection**	**Number of studies**	**Number of prevalence measures**	**Total sample size**	**Prevalence range of low stress (%)** ***n*** **= 12**	**Prevalence range of moderate stress (%)** ***n*** **= 12**	**Prevalence range of high stress (%)** ***n*** **= 14**
**All**	2008–2019	13	38	2804	0.8–65	5.9–84.5	6.7–99.2
**Males and females**	2014–2019	9	28	2298	0.8–52	26–84.5	12.2–99.2
**Females only**	2008–2017	4	10	506	13.6–65	5.9–63.6	6.7–94.1
**Nursing students in clinical training**	2015–2017	4	10	559	52–65	5.9–28.3	6.7–94.1
**Nursing students in all years**	2008–2019	9	28	2245	0.8–59.8	43.5–84.5	12.2–99.2
**Used validated tools**	2008–2019	12	36	2252	3.3–65	5.9–84.5	6.7–94.1
**Used non-validated tools**	20014–2015	1	2	128	0.8	–	99.2
**Saudi Arabia**							
**Groups**	**Year of data collection**	**Number of studies**	**Number of prevalence measure**	**Total sample size**	**Prevalence range of low stress (%)** ***n*** **= 3**	**Prevalence range of moderate stress (%)** ***n*** **= 5**	**Prevalence range of high stress (%)** ***n*** **= 5**
**All**	2015–2017	5	13	280	5.2–65	5.9–75.3	6.7–94.1
**Males and females**	2015–2016	2	5	147	5.2	28–75.3	19.6–72
**Females only**	2017	3	8	133	13.6–65	5.9–63.6	6.7–94.1
**Egypt**							
**Groups**	**Year of data collection**	**Number of studies**	**Number of prevalence measure**	**Total sample size**	**Prevalence range of low stress (%)** ***n*** **= 6**	**Prevalence range of moderate stress (%)** ***n*** **= 5**	**Prevalence range of high stress (%)** ***n*** **= 6**
**All**	2008–2019	5	17	1925	10.1–59.8	26–65	21.3–46.4
**Males and Females**	2014–2019	4	15	1552	10.1–52	26–65	21.3–46.4
**Females only**	2008–2009	1	2	373	59.8	–	40.2
**Jordan**							
**Groups**	**Year of data collection**	**Number of studies**	**Number of prevalence measure**	**Total sample size**	**Prevalence range of low stress (%)** ***n*** **= 1**	**Prevalence range of moderate stress (%)** ***n*** **= 1**	**Prevalence range of high stress (%)** ***n*** **= 1**
**Males and females**	–	1	3	271	3.3	84.5	12.2
**Iraq**							
**Groups**	**Year of data collection**	**Number of studies**	**Number of prevalence measure**	**Total sample size**	**Prevalence range of low stress (%)** ***n*** **= 2**	**Prevalence range of Moderate stress (%)** ***n*** **= 1**	**Prevalence range of high stress (%)** ***n*** **= 2**
**Males and females**	2018	2	5	328	0.8–22	65	13.0–99.2

*n* Number of individual primary studies

Some studies reported significantly higher stress levels in nursing students living in rural areas [70], having a father with low school education or non-professional background (e.g., farmers or manual workers) [70], low grades in the previous year [70], low family income [71, 72], enrolled in community courses [73, 74], spending six or more hours studying per day [72], 6 h or less of sleep per night [72], and suffering from overweight and obesity [71] (Table S4). The impact of age [71, 75–78], gender [71, 76, 79, 80], marital status [77, 78], stages/levels of student’s study [77–83], and student’s interest in nursing [74, 75], on stress levels seems to be inconsistent (Table S4).

### Overview of studies with data on stressors

Table 2 summarizes the various types of stressors reported among nursing students. A total of 36 primary studies reported data on stressors among nursing students in Saudi Arabia, Egypt, Jordan, Iraq, Pakistan, Oman, Palestine, and Bahrain: 26 reported data on stressors during the clinical period, 15 during the academic period, and in 15, the exact academic or clinical period could not be determined. In addition to stressors during the clinical and academic periods, studies also identified stressors ‘external’ to the training periods. We grouped the external stressors to be related to the ‘physical environment’ or being ‘intrapersonal.’

**Table 2:** Summary of reported stressors among nursing students in Middle East and North Africa (MENA) countries

Category of stressors	Specific stressors (number of primary studies)
**Clinical training period (*n*** **= 26)**	
**Stress from patient care (*n*** **= 19)**	Lack of experience and ability in providing nursing care and in making judgments (*n* = 7) [84–89]
	Do not know how to help patients with physio-psycho-social problems (*n* = 3) [84, 85, 90]
	Unable to reach one’s expectations (*n* = 0)
	Unable to provide appropriate responses to doctors’, teachers’, and patients’ questions (*n* = 1) [84]
	Worry about not being trusted or accepted by patients or patients’ family (*n* = 1) [90]
	Unable to provide patients with good nursing care (*n* = 0)
	Do not know how to communicate with patients (*n* = 2) [84, 90]
	Experience difficulties in changing from the role of a student to that of a nurse (*n* = 1) [76]
	Unspecified (*n* = 14) [72, 81–84, 90–98]
**Stress from teachers and nursing staff (*n*** **= 16)**	Experience discrepancy between theory and practice (*n* = 3) [85, 88, 90]
	Do not know how to discuss patients’ illness with teachers, and medical and nursing personnel (*n* = 1) [85]
	Feel stressed that teacher’s instruction is different from one’s expectations (*n* = 0)
	Medical personnel lack empathy and are not willing to help (*n* = 3) [86–88]
	Feel that teachers do not give fair evaluation on students (*n* = 0)
	Lack of care and guidance from teachers (*n* = 1) [89]
	Not a specific stressor (Stress from teachers and nursing staff (*n* = 12) [72–74, 81, 85, 89, 91, 92, 95–98])
**Stress from assignments and workload (*n*** **= 23)**	Worry about bad grades (*n* = 2) [85, 98]
	Experience pressure from the nature and quality of clinical practice (*n* = 2) [84, 99]
	Feel that one’s performance does not meet teachers’ expectations (*n* = 2) [84, 85]
	Feel that the requirements of clinical practice exceed one’s physical and emotional endurance (*n* = 3) [84, 90, 100]
	Feel that dull and inflexible clinical practice affects one’s family and social life (*n* = 1) [78]
	Not a specific stressor (stress from assignments and workload (*n* = 20) [72–75, 81–89, 91, 92, 95–98, 101]
**Stress from peers and daily life (*n*** **= 7)**	Experience competition from peers in school and clinical practice (*n* = 0)
	Feel pressure from teachers who evaluate students’ performance by comparison (*n* = 0)
	Feel that clinical practice affects one’s involvement in extracurricular activities (*n* = 0)
	Cannot get along with other peers in the group (*n* = 1) [93]
	Not a specific stressor (stress from peers and daily life (*n* = 6) [72–74, 86, 92, 101])
**Stress from lack of professional knowledge and skills (*n*** **= 12)**	Unfamiliar with medical history and terms (*n* = 0)
	Unfamiliar with professional nursing skills (*n* = 0)
	Unfamiliar with patients’ diagnoses and treatments (*n* = 2) [87, 88]
	Not a specific stressor (stress from lack of professional knowledge and skills (*n* = 10) [72, 73, 75, 81–83, 90, 92, 98, 101])
	Feel stressed in the hospital environment where clinical practice takes place (*n* = 11) [72, 75, 81, 82, 84–86, 92, 95, 98]
	Unfamiliar with the ward facilities (*n* = 1) [85]
	Feel stressed from the rapid change in patient’s condition (*n* = 0)
	Unspecified (*n* = 2) [78, 84]
**Academic period (*n*** **= 15)**	
**Academic sources (*n*** **= 15)**	Examination load (*n* = 4) [87, 88, 102, 103]
	Course load (*n* = 5) [76, 87, 88, 102, 103]
	Lack of enough break time; not enough leisure time (*n* = 7) [77, 82, 87, 88, 93, 102, 103]
	Getting lower grade than anticipated (*n* = 6) [77, 86–88, 93, 100]
	Imbalance between leisure and study time (*n* = 4) [86–88, 93]
	Unable to enjoy study (*n* = 1) [93]
	Inconsiderate and insensitive instructors (*n* = 2) [87, 88]
	Being in 4th year (*n* = 2) [100, 101]
	Unspecified (academic sources (*n* = 5) [87, 88, 99, 103, 104], academic workload (*n* = 5) [70, 77, 78, 82, 93])
**Stressors external to training periods (*n*** **= 15)**	
**Physical environmental sources (*n*** **= 11)**	External environment (*n* = 1) [82]
	Accommodation problems (*n* = 1) [70]
	Congested classroom (*n* = 3) [70, 87, 88]
	Living environmental change (*n* = 2) [78, 93]
	Inadequate safety and security (*n* = 3) [86, 90, 93]
	Lack of recreation facilities (*n* = 4) [86–88, 93]
	Absence of calm environment (*n* = 3) [87, 88, 93]
	Inadequate water provision (*n* = 1) [93]
	Long waits to get service (*n* = 1) [77]
	College environment (*n* = 1) [76]
	Moving location (*n* = 1) [92]
	Noise (*n* = 2) [90, 92]
	Transportation difficulties (*n* = 2) [87, 88]
	Smell and unfavorable odors (*n* = 1) [90]
**Intrapersonal sources (*n*** **= 14)**	Social/personal environment (*n* = 2) [76, 92]
	Lack of confidence and inability to make decisions (*n* = 2) [87, 88]
	Anxiety and depression (*n* = 1) [70]
	Feeling of homesickness (*n* = 1) [93]
	Change in sleeping patterns/not enough sleep (*n* = 5) [77, 78, 87, 88, 93]
	Changes in eating habits (*n* = 1) [78]
	New responsibilities (*n* = 1) [78]
	Personal illnesses (*n* = 4) [87, 88, 92, 93]
	Financial problems (*n* = 3) [87, 88, 100]
	Anticipation of graduation (*n* = 1) [78]
	Important decisions about future career (*n* = 7) [70, 77, 81, 86–88, 102]
	Not well defined (Intrapersonal sources (*n* = 2) [99, 105])

Clinical training stressors were classified as per the Perceived Stress Scale (PSS) [61]

*n* Number of individual primary studies

Based on the total number of primary studies, stressors related to clinical training are most commonly reported among nursing students followed by academic stressors. The predominantly reported specific stressors during clinical training are associated with assignments and workload (e.g., pressure from the nature and quality of clinical practice, a feeling that requirements of clinical practice are exceeding their physical and emotional endurance) and patient care (e.g., lack of experience and ability in providing nursing care and in making judgments, ‘do not know how to help patients with physio-psycho-social problems’). Lack of break/leisure time, getting lower grades than anticipated, and examination and course load are the main specific sources of academic stressors. External stressors related to the physical environment include lack of recreation facilities; absence of a calm, safe, and secure environment; and congested classrooms. External stressors related to intrapersonal reasons reported by the nursing students include uncertainty about their future career, change in sleep pattern, and financial problems.

No significant differences are observed in the type and level of stressors between students in private nursing schools and those in public schools [79] or according to the place of residence [84, 90], age [79, 84, 90], gender [84, 90], religion [84], marital status [79, 90], and grade point average (GPA) of the last term (Table S4).

### Overview of studies with data on stress coping strategies

Table 3 summarizes the stress coping strategies that nursing students use to deal with stress. A total of 23 primary studies reported data on stress coping strategies used by nursing students in Saudi Arabia, Egypt, Jordan, Oman, Pakistan, and Sudan: 19 studies report the use of problem-focused coping, 20 studies the use of emotion-focused coping, and 17 studies the use of dysfunctional coping. The most widely used problem-focused stress coping strategies are active coping (e.g., problem understanding and solving) and seeking social support for instrumental reasons (e.g., asking others for help and developing social support). Whereas, positive reinforcement and growth (e.g., staying optimistic and wishful thinking) and turning to religion (e.g., use of religion, prayer, invocation, and finding comfort in religion or spiritual beliefs) are the most widely used emotion-focused stress coping strategies. The most commonly used forms of dysfunctional coping strategies are mental disengagement (e.g., transference, become involved in other activities) and behavioral disengagement (e.g., avoidance, social withdrawal).

**Table 3:** Summary of reported coping mechanisms and strategies among nursing students in Middle East and North Africa (MENA) countries

Coping mechanism (number of primary studies)	Coping strategy	Specific coping strategy (primary studies)
**Problem-focused (*n*** **= 19)**	Active coping	Problem solving (*n* = 11) [70, 72, 74, 75, 81, 85, 91, 92, 95, 97, 98]
		Taking action to try to make the situation better (*n* = 2) [106, 107]
		Problem-focused coping (*n* = 1) [108]
		Problem understanding and solving (*n* = 2) [107, 109]
		Solving family problems (*n* = 1) [110]
		Self-reliance (*n* = 1) [110]
		Did what is expected of me (*n* = 1) [76]
		Tension reduction (*n* =1) [108]
		Self-analysis to understand the situation better (*n* = 1) [76]
		Unspecified (*n* = 1) [111]
	Planning	Planning strategy (*n* = 1) [71]
	Suppression of competing activities	Engaging in demanding activities (*n* = 1) [110]
	Restraint coping	Passive coping (*n* = 1) [94]
	Seeking social support for instrumental reasons	Asking others for help (*n* = 1) [109]
		Seeking diversions (*n* = 1) [110]
		Developing social support (*n* = 1) [110]
**Emotion-focused (*n*** **= 20)**	Seeking social support for emotional reasons	Unspecified (*n* = 1) [108]
		Discuss feeling with friends or classmates (*n* = 1) [76]
	Positive reinforcement and growth	Positive reframing (*n* = 2) [71, 107]
		Focusing on the positive (*n* = 1) [108]
		Wishful thinking (*n* = 3) [73, 107, 108]
		Staying optimistic (*n* = 11) [72, 74, 75, 81, 83, 85, 92, 95, 97, 98, 112]
	Acceptance	Unspecified (*n* = 2) [71, 111]
		Accept the situation (*n* = 2) [69, 76]
	Turning to religion	Practicing religion (*n* = 1) [111]
		Prying or mediating (*n* = 1) [106]
		Finding comfort in religion or spiritual beliefs (*n* = 1) [106]
		Religious related strategies come first (*n* = 1) [71]
		Use of religion, prayer, invocation (*n* = 2) [69, 109]
**Dysfunctional coping (*n*** **= 17)**	Focus on and venting of emotions	Venting (*n* = 1) [71]
		Show their feelings and express their reaction (*n* = 2) [107, 109]
		Ventilating feelings (*n* = 1) [110]
		Self-blaming (*n* = 1) [71]
		Self-criticism (*n* = 1) [73]
	Behavioral disengagement	Unspecified (*n* = 1) [71]
		Social withdrawal (*n* = 1) [73]
		Avoidance (*n* = 9) [72–74, 85, 92, 97, 98, 107, 112]
	Mental disengagement	Become involved in other activities (*n* = 1) [76]
		Detachment (*n* = 1) [108]
		Transference (*n* = 9) [72, 74, 75, 83, 92, 95, 97, 98, 112]
		Physical exercise and relaxation (*n* = 1) [109]
	Denial	Unspecified (*n* = 1) [71]

The coping mechanisms were classified as per the COPE inventory tool [62, 113, 114]

*n* Number of individual primary studies

The reported relationship between stress levels and the used coping strategies is inconsistent. Two included studies [81, 91] suggest a significantly negative correlation between the total PSS score (stress level) and the use of specific coping strategies, namely problem-solving. Only one study reported higher stress levels among students who utilized coping strategies like avoidance or transference strategies [100].

Some included studies suggested differences in the type of specific coping strategies used according to gender [71, 85], academic level [71], and living with family or alone [85]. The coping strategies used by the students also varied according to the stressor. During clinical training, students experiencing stress from assignments and patient care, peers, daily life, teachers, and nursing staff were found to frequently use avoidance [85, 92], transference [92], problem solving [85, 92], and staying optimistic [92]. Common external (physical environmental or intrapersonal) stressors were linked to the use of transference [92], problem solving [92], and staying optimistic [92].

### Overview of stress management recommendations for nursing students, nursing faculty and educators, and nursing institutions

Recommendations reported in the included systematic reviews on how nursing students, nursing faculty and educators, and nursing institutions can aim to reduce stress levels, and manage stressors to maximize knowledge gain and productivity among nursing students are synthesized (Table 4).

**Table 4:** Reported stress management recommendations for nursing students, nursing faculty and educators, and nursing institutions in Middle East and North Africa (MENA) countries

Stress management recommendations		
For nursing institutions	For nursing students	For nursing faculty and educators
▪ Establish a student support system through which the students can be equipped with effective coping strategies [64, 65, 115, 116]. This includes the following: ◦ A structured mentorship program to effectively reduce psychological stress and enhance nursing students' sense of self-confidence and psychological sense of belonging [63, 67, 86, 117, 118] and to adequately manage and regulate the academic and clinical practice stressor [69]. ◦ Trainings on self-efficacy [69], stress management, time management, counseling, and coping skills enhancement [63, 64, 67, 69, 118–120]. ◦ A structured orientation by nursing school administrators [69, 86, 100]. ◦ Stress interventions which are theory-driven such as relaxation and cognitive appraisal techniques [67, 69]. ◦ The use of multifaceted strategies such as peer and staff mentorship, provision of social support and professional networks, creating a caring learning environment, positive faculty role modeling utilizing positive coping, and proactive learning [67, 121]. ◦ Follow-through to nurses in the profession to incorporate methods to cope with stress and develop effective coping strategies. Hospital administrators and other stakeholders may benefit with the continued process from nursing education to entry and beyond for the nurse [69]. ▪ Emphasize regular training sessions and workshops for nurse educators aiming to enhance their communication, social, and interpersonal skills to assist in dealing with students [69] and enable them to work effectively with students [65, 97, 122, 123]. ▪ Linking specific coping strategies to nursing school stressors is helpful to better prepare nursing students, while managing stress effectively [63, 112, 124]. This will allow teachers to support their students more effectively, which in turn may result in improving clinical nursing education [65, 124]. ▪ Hospital administrators should promote policies that facilitate a training environment in which students are supported and inspired while they engage in clinical practice [125] and develop continuous professional education programs for their staff so they can learn how to appropriately deal with students [64, 126].	▪ Utilization of positive coping mechanisms reduces stress levels in nursing students and can impact the effects of stress on their physiological and psychological well-being [67]. ▪ Nursing students should be aware of the significance of using problem-solving approaches, as well as understand that combination of problem-solving techniques can alleviate stress levels [67]. ▪ Establishment of a social support unit/centre such as family, friends, and relatives would be an asset to counter the adverse effects of stress [69, 127, 128].	▪ Mentor students to develop and strengthen problem-based, rather than emotion-based behavior to cope with stress [65]. ▪ Provide a supportive clinical learning environment and strengthen positive-coping mechanisms in students, to better deal with stressors and maximize clinical learning [63, 69, 119, 129–131]. ▪ Formulate custom-tailored coping strategies and interventions by identifying coping factors/predictors to lessen, reduce and prevent stress in order to facilitate maximum learning both in the theoretical and clinical setting [63]. ▪ Pay more attention to nursing students with high stress levels and offer adequate support as this is critical for their successful completion of the courses [67]. ▪ Address students’ need to handle stressors effectively. Give more attention to clinical training, minimize the required paper work, prepare all professionals involved in training nursing students adequately, and offer simulation practice to enable the students to provide patient care prior to entering the actual clinical context [97]. ▪ Encourage students to discuss their feelings and stressors in order to provide appropriate interventions [64, 132]. ▪ Include video films about clinical settings, invite expert guest speakers and host frequent field visits (during orientation period) to decrease initial clinical stress [64, 132]. ▪ Plan strategies to prevent stress recurrence among nursing students during clinical training while keeping them driven to achieve maximum knowledge [63].

## Discussion

Our overview synthesizes the evidence on nursing students in the MENA countries about perceived stress, stressors, and the stress coping strategies utilized by them to manage stress. We reviewed 7 systematic reviews and 42 primary studies that include data from nine the MENA countries namely, Bahrain, Egypt, Iraq, Jordan, Oman, Pakistan, Palestine, Saudi Arabia, and Sudan. Prevalence data from the majority of studies suggest that moderate and high stress levels predominate among nursing students in the region. Differences related to gender, training period, or the type of tool used to measure stress remain unclear given the wide variability in the reported prevalence measures across all stress levels. Commonly reported stressors among nursing students are related to clinical training (assignments, workload, and patient care), and academic training (lack of break/leisure time, grades, and examination and course load). Studies report the utilization of three predominant stress coping mechanisms: problem-focused (dealing with the problem), emotion-focused (regulating the emotion), and dysfunctional coping (venting the emotions). The most commonly utilized strategies within the problem-focused mechanism include active coping which in turn includes, specific strategies namely “problem understanding and solving” and “seeking social support.” Similarly, within emotion-focused mechanism, positive reinforcement and growth strategy, which includes “staying optimistic” and “wishful thinking” were more common. In the case of dysfunctional coping mechanism, behavioral, and mental disengagement, “avoidance” and “transference” were the most commonly used strategies and specific strategies respectively.

### Variation in perceived stress prevalence data and comparison with international data

While moderate stress levels are reported in studies conducted in China [133], Hong Kong [134], and Nepal [135], our findings suggest that the stress level among nursing students in the MENA region ranges from moderate to high. The prevalence range of stress levels similar to that observed in our study has also been found in medical students in the region [36, 40, 41, 46, 136, 137], internationally [34, 37, 38, 43, 45, 138], and among midwifery students [66]. The wide variability in the stress prevalence measures found in our review could be explained by certain characteristics of the tools used. Some questionnaires used to measure stress levels in the included studies evaluate stress during the previous month [139] and some others during an undetermined period [71, 99, 140]. Also, certain questionnaires used are designed to measure stress levels in any life situation [139] and some others have been adapted to be used among nursing students [71, 72, 93, 99, 140]. The wide range of prevalence measures across all levels of stress could be also explained by the limited sample size of the primary studies and representativeness of the selected students. Some evidence shows increased levels of stress as the nursing students progress in their educational program [102, 141], whereas, some other studies conclude no change in the stress level [129] between the academic years [135].

### Stressors

Similar to our findings, studies conducted in non-MENA countries have reported clinical training stressors, particularly clinical assignments and workload, as the most common stressors among nursing students [133–135]. Specifically, we also found that patient care is a common stressor for nursing students during their clinical training. Procedures related to patient care, examination frequency, and the amount of overall workload during clinical training must be revisited.

A global systematic review excluding the MENA concluded that the most common stressors among nursing students are academic stressors (workload and problems associated with studying) followed by clinical stressors (such as fear of unknown situations, mistakes with patients or handling of technical equipment) [129]. This can be explained by the predominance of studies including nursing students in the preparatory and preclinical years (years 1–2) which are generally characterized by more academic workload than clinical years (years 3–4) in this systematic review [129].

Most of the published literature focuses on assessing academic and clinical stressors. The importance and potential impact of stressors external to training periods, such as the physical environment and intrapersonal stressors have been less studied. Likewise, the lack of a standardized approach to categorize stressors in these studies makes it difficult to compare results between studies. The grouping of stressors in this overview may be useful for future research on this topic. Researchers may choose to assess stressors based on academic, clinical, or factors external to training.

### Data on the use of coping strategies

The wide variation found in coping strategies utilized by nursing students in the MENA countries and worldwide [133–135, 142] can be explained by the differences in cultural, socio-economic, and geographic contexts [142]. Comparable to our findings, emotional (e.g., expressions of empathy) and instrumental social support (e.g., tangible aid and service), and religion are identified to be commonly used stress coping mechanisms by nursing students in Hong Kong [134] and Malaysia [142]. Other dysfunctional coping strategies, such as the use of alcohol or illicit drugs were not assessed in the included studies.

The relationship between socio-economic factors and stress levels or stressors could not be confirmed, as most of the included primary studies are cross-sectional and not designed to assess causal associations. A possible link between stress levels and factors, such as gender [90] and living with family or alone [85] can be established on the basis of studies included in our review.

### Relationship between stress levels, stressors, and coping strategies

Published literature indicates a potential link between stressors and the coping strategies utilized by nursing students [71, 85]. A recent study demonstrated a statistically significant correlation of the six domains of stressors during clinical practice comprising of patient care, clinical educators/instructors and ward staff, clinical assignments and workload, peers and nursing students from other colleges, lack of professional knowledge and skills, and the clinical environment) with coping strategies [142]. Available data on the relationship between stress levels and the used coping strategies is limited and inconsistent [81, 91, 100]. Data from another study, however, suggests that the use of optimism, self-efficacy, and resilient coping by nursing students can have an impact on their perceived stress [37]. Additional studies designed to assess these potential associations are needed to establish the evidence.

### Recommendations for stress management

To manage stress among nursing students, it is highly recommended by several published studies that nursing institutions must recognize their role in improving stress management [63–65, 67, 69, 86, 100, 115–121]. Nursing institutions are encouraged to provide a supportive clinical learning environment and to establish a strong support system to equip both nursing students and educators with effective coping strategies. Although evidence is absent about the type of intervention that would be effective to reduce excessive stress among nursing students, some statistically significant effect was found for interventions which focused on reducing the number or intensity of stressors through curriculum revision or improving students’ coping response by indulging in art therapy and biofeedback-assisted relaxation training [143].

### Strengths and limitations

To our knowledge, this is the most comprehensive, up-to-date, systematic overview synthesizing several dimensions of stress and coping behavior in a key population of the health care system for the MENA region. We searched multiple grey and non-grey literature sources for systematic reviews and primary studies to provide comprehensive evidence on the epidemiology of perceived stress among nursing students in the region. This compilation of evidence will serve as a benchmark for nursing students, nursing faculty and educators, and nursing institutions to help direct future interventions to optimize learning and prepare nursing students to manage stress effectively. Moreover, most of the included primary studies (12 out of 13) with prevalence data utilized a validated tool to measure the prevalence of perceived stress which minimizes the bias from the measurement tool. Most of the validated tools utilized in the studies are designed to assess clinical stressors in nursing students [61, 90, 119, 140, 144–146].

Some included systematic reviews had specific inclusion criteria, such as, nursing students undergoing clinical training only or with a certain standard of methodological quality, which could explain the limited number of included primary studies in the systematic reviews. Moreover, none of the included systematic reviews have included studies published in a language other than English which could have led to an incomplete selection of primary studies relevant for the MENA countries. Out of the seven included systematic reviews, five had global geographical coverage and did not search grey literature or specific sources relevant for countries of the MENA region. Despite our best efforts to include all available data, data on the topic in other country-specific grey literature sources could exist. The included systematic reviews have searched data up to August 2018. In the absence of recent systematic reviews and to complete the collected data with recent studies published in the past 2 years, we conducted a hand search of primary studies in grey and non-grey literature sources. Despite this updated search, other recent primary studies could have been missed. Out of the seven included systematic reviews, five [63, 65, 66, 68, 69] were found through a hand search. This is explained by the fact that none of the hand searched systematic reviews have mentioned a term related to the MENA countries in the searchable fields of the used data platforms.

## Conclusions

Our findings suggest that the stress level among nursing students in the MENA region ranges from moderate to high. The limited data on stress prevalence among nursing students in all the MENA countries prevents the estimation of its magnitude with certainty; hence, comparability of stress prevalence between countries and other regions is also not possible. Differences due to gender, clinical training period, or type of tool used remain unclear given the wide variability in the reported prevalence measures across all stress levels. Nursing students commonly report stressors related to both clinical and academic training components of the nursing curriculum. Studies report an equal utilization of three predominant stress coping strategies by the nursing students: problem focused (dealing with the problem), emotion focused (regulating the emotion), and dysfunctional (venting the emotions). The link between stressors, and coping strategies and stress levels remains unclear. Although the significance of using the problem-solving approach to manage stress is well-established, there is a need to identify effective strategies to reduce excessive stress and increase the utility of positive coping strategies. Nursing institutions should establish a strong support system for students and educators to equip them with effective coping strategies. Reducing the number or intensity of stressors through curriculum revision and improving students’ coping response could contribute to the reduction of stress levels among students. Nursing faculty and educators are encouraged to mentor students to develop and strengthen problem-based, rather than emotion-based behavior to cope with stress and to provide a supportive clinical learning environment. While stress may not be preventable, it appears coping with stressors especially during the clinical training of the nursing curriculum is essential to maximize knowledge gain and productivity and prevent burnout among nursing students.

## Supplementary Information

**Additional file 1: Table S1.** The 2009 PRISMA checklist for reporting a systematic review. **Table S2.** PRIO-harms checklist for reporting an overview of systematic reviews (OoSRs). **Table S3.** Characteristics of the included systematic reviews. **Table S4.** Prevalence of stress, stressors and coping strategies among nursing students in the MENA countries with available data. **Table S5.** Quality assessment of included systematic reviews. **Table S6.** Quality assessment of included studies.

## Data Availability

The datasets generated during and/or analyzed during the current study are available from the corresponding author on reasonable request.

## Abbreviations

PRISMA: Preferred Reporting Items for Systematic Reviews and Meta-Analyses
PRIO-harms: The Preferred Reporting Items for Overviews of Systematic Reviews
MENA: Middle East and North Africa
UAE: The United Arab Emirates
ROB: Risk of bias
PSS: Perceived Stress Scale
SAS: Stress Assessment Scale
PPSS: Physio-Psychosocial Stress Scale
SINS: Stressors in Nursing Students Scale
SSS: Student Stress Survey
SSCI: Students Stress and Coping Inventory
SCSS: Student Clinical Stressor Scale
SNSI: Student Nurse Stress Index
CBI: Coping Behaviors Inventory
COPE: Coping Orientation to Problems Experienced
ACOPE: Adolescent Coping Orientation for Problem Experiences
RWCSQ: Revised Ways of Coping Strategies Questionnaire
SSCI: Students Stress and Coping Inventory

## References

[1] Barry S, Ward L (2017). Undergraduate nursing students’ understandings of mental health: a review of the literature. Issues Ment Health Nurs.

[2] Moreira DP, Furegato ARF (2013). Stress and depression among students of the last semester in two nursing courses. Rev Lat Am Enfermagem.

[3] Rudman A, Gustavsson JP (2012). Burnout during nursing education predicts lower occupational preparedness and future clinical performance: a longitudinal study. Int J Nurs Stud.

[4] Tung YJ, Lo KKH, Ho RCM, Tam WSW (2018). Prevalence of depression among nursing students: A systematic review and meta-analysis. Nurse Educ Today.

[5] Phillips A (1989). Nursing Education in Saudi Arabia. Ann Saudi Med.

[6] The American Association of Colleges of Nursing (AACN). Nursing Education Programs / Baccalaureate Education. 2020. Available from: https://www.aacnnursing.org/Nursing-Education-Programs/Baccalaureate-Education. Accessed 20 May 2020.

[7] Moffat KJ, McConnachie A, Ross S, Morrison JM (2004). First year medical student stress and coping in a problem-based learning medical curriculum. Med Educ.

[8] Elliott M (2002). The clinical environment: a source of stress for undergraduate nurses. Aust J Adv Nurs.

[9] Newbury-Birch D, Walshaw D, Kamali F (2001). Drink and drugs: from medical students to doctors. Drug Alcohol Depend.

[10] Ashton CH, Kamali F (1995). Personality, lifestyles, alcohol and drug consumption in a sample of British medical students. Med Educ.

[11] Woloschuk W, Harasym PH, Temple W (2004). Attitude change during medical school: a cohort study. Med Educ.

[12] Gibbons C (2010). Stress, coping and burn-out in nursing students. Int J Nurs Stud.

[13] Xie Z, Wang A, Chen B (2011). Nurse burnout and its association with occupational stress in a cross-sectional study in Shanghai. J Adv Nurs.

[14] Mészáros V, Cserháti Z, Oláh A, Perczel Forintos D, Adám S (2013). Coping with work-related stress in health care professionals -- strategies for the prevention of burnout and depression. Orv Hetil.

[15] Institute for Health Metrics and Evaluation (IHME) (2018). Findings from the global burden of disease study 2017.

[16] World Health Organization (2017). Depression and other common mental disorders: global health estimates.

[17] Ferrari AJ, Somerville AJ, Baxter AJ, Norman R, Patten SB, Vos T, Whiteford HA (2013). Global variation in the prevalence and incidence of major depressive disorder: a systematic review of the epidemiological literature. Psychol Med.

[18] Young CB, Fang DZ, Zisook S (2010). Depression in Asian–American and Caucasian undergraduate students. J Affect Disord.

[19] Buchanan JL (2012). Prevention of depression in the college student population: a review of the literature. Arch Psychiatr Nurs.

[20] Steptoe A, Tsuda A, Tanaka Y, Wardle J (2007). Depressive symptoms, socio-economic background, sense of control, and cultural factors in university students from 23 countries. Int J Behav Med.

[21] Olvera Alvarez H, Provencio-Vasquez E, Slavich G, Laurent J, Browning M, McKee-Lopez G (2019). Stress and health in nursing students. Nurs Res.

[22] Shukri R (2005). Status of nursing in the Arab world. Ethn Dis.

[23] Sheikh JI, Cheema S, Chaabna K, Lowenfels AB, Mamtani R (2019). Capacity building in health care professions within the Gulf cooperation council countries: paving the way forward. BMC Med Educ.

[24] Courage MM, Godbey KL (1992). Student retention: policies and services to enhance persistence to graduation. Nurse Educ.

[25] Smith-Wacholz HC, Wetmore JP, Conway C, McCarley M (2019). Retention of nursing students: an integrative review. Nurs Educ Perspect.

[26] Lindop E (1989). Individual stress and its relationship to termination of nurse training. Nurse Educ Today.

[27] Lindop E (1987). Factors associated with student and pupil nurse wastage. J Adv Nurs.

[28] Shikai N, Uji M, Chen Z, Hiramura H, Tanaka N, Shono M, Kitamura T (2007). The role of coping styles and self-efficacy in the development of dysphoric mood among nursing students. J Psychopathol Behav Assess.

[29] Harrington R, Fudge H, Rutter M, Pickles A, Hill J (1990). Adult outcomes of childhood and adolescent depression: I. Psychiatric status. Arch Gen Psychiatry.

[30] Hofstra MB, Van der Ende JAN, Verhulst FC (2000). Continuity and change of psychopathology from childhood into adulthood: a 14-year follow-up study. J Am Acad Child Adolesc Psychiatry.

[31] Kim-Cohen J, Caspi A, Moffitt TE, Harrington H, Milne BJ, Poulton R (2003). Prior juvenile diagnoses in adults with mental disorder: developmental follow-back of a prospective-longitudinal cohort. Arch Gen Psychiatry.

[32] Hofstra MB, Van Der Ende JAN, Verhulst FC (2002). Child and adolescent problems predict dsm-iv disorders in adulthood: a 14-year follow-up of a dutch epidemiological sample. J Am Acad Child Adolesc Psychiatry.

[33] Deary IJ, Watson R, Hogston R (2003). A longitudinal cohort study of burnout and attrition in nursing students. J Adv Nurs.

[34] Voltmer E, Kötter T, Spahn C (2012). Perceived medical school stress and the development of behavior and experience patterns in German medical students. Med Teach.

[35] Castaldelli-Maia JM, Lewis T, Marques dos Santos N, Picon F, Kadhum M, Farrell SM, et al. Stressors, psychological distress, and mental health problems amongst Brazilian medical students. Int Rev Psychiatry. 2019;31:7–8, 603–7. 10.1080/09540261.2019.1669335 31612743

[36] Shawi AFA, Abdullateef AN, Khedher MA, Rejab MS, Khaleel RN (2018). Assessing stress among medical students in Anbar governorate, Iraq: a cross-sectional study. Pan Afr Med J.

[37] Heinen I, Bullinger M, Kocalevent R-D (2017). Perceived stress in first year medical students - associations with personal resources and emotional distress. BMC Med Educ.

[38] Melaku L, Mossie A, Negash A (2015). Stress among medical students and its association with substance use and academic performance. J Biomed Educ.

[39] Ludwig AB, Burton W, Weingarten J, Milan F, Myers DC, Kligler B (2015). Depression and stress amongst undergraduate medical students. BMC Med Educ.

[40] Abdulghani HM, AlKanhal AA, Mahmoud ES, Ponnamperuma GG, Alfaris EA (2011). Stress and its effects on medical students: a cross-sectional study at a college of medicine in Saudi Arabia. J Health Popul Nutr.

[41] Abdulghani HM (2008). Stress and depression among medical students: a cross sectional study at a Medical College in Saudi Aarabia. Pak J Med Sci.

[42] Dahlin M, Joneborg N, Runeson B (2005). Stress and depression among medical students: A cross-sectional study. Med Educ.

[43] Sherina MS, Rampal L, Kaneson N (2004). Psychological stress among undergraduate medical students. Med J Malaysia.

[44] Kiessling C, Schubert B, Scheffner D, Burger W (2004). First year medical students’ perceptions of stress and support: a comparison between reformed and traditional track curricula. Med Educ.

[45] Esan O, Esan A, Folasire A, Oluwajulugbe P. Mental health and wellbeing of medical students in Nigeria: a systematic review. Int Rev Psychiatry. 2019:1–12. 10.1080/09540261.2019.1677220 31646912

[46] Elzubeir MA, Elzubeir KE, Magzoub ME (2010). Stress and coping strategies among Arab medical students: towards a research agenda. Educ Health (Abingdon).

[47] Witt K, Boland A, Lamblin M, McGorry PD, Veness B, Cipriani A (2019). Effectiveness of universal programmes for the prevention of suicidal ideation, behaviour and mental ill health in medical students: a systematic review and meta-analysis. Evid Based Ment Health.

[48] Doraiswamy S, Jithesh A, Chaabane S, Abraham A, Chaabna K, Cheema S (2020). Perinatal mental illness in the Middle East and North Africa Region-a systematic overview. Int J Environ Res Public Health.

[49] Chaabna K, Cheema S, Abraham A, Alrouh H, Lowenfels AB, Maisonneuve P, Mamtani R (2018). Systematic overview of hepatitis C infection in the Middle East and North Africa. World J Gastroenterol.

[50] Chaabane S, Chaabna K, Abraham A, Mamtani R, Cheema S (2020). Physical activity and sedentary behaviour in the Middle East and North Africa: an overview of systematic reviews and meta-analysis. Sci Rep.

[51] Chaabna K, Cheema S, Abraham A, Alrouh H, Mamtani R, Sheikh JI (2018). Gray literature in systematic reviews on population health in the Middle East and North Africa: protocol of an overview of systematic reviews and evidence mapping. Syst Rev.

[52] Becker L, Oxman A (2011). Overviews of reviews. Cochrane handbook for systematic reviews of interventions version 510.

[53] Moher D, Liberati A, Tetzlaff J, Altman DG (2009). Preferred reporting items for systematic reviews and meta-analyses: the PRISMA statement. J Clin Epidemiol.

[54] Bougioukas KI, Liakos A, Tsapas A, Ntzani E, Haidich A-B (2018). Preferred reporting items for overviews of systematic reviews including harms checklist: a pilot tool to be used for balanced reporting of benefits and harms. J Clin Epidemiol.

[55] Higgins J, Green S (2011). Cochrane handbook for systematic reviews of interventions version 5.1.0.

[56] Ouzzani M, Hammady H, Fedorowicz Z, Elmagarmid A (2016). Rayyan—a web and mobile app for systematic reviews. Syst Rev.

[57] Shea BJ, Grimshaw JM, Wells GA, Boers M, Andersson N, Hamel C, Porter AC, Tugwell P, Moher D, Bouter LM (2007). Development of AMSTAR: a measurement tool to assess the methodological quality of systematic reviews. BMC Med Res Methodol.

[58] Higgins JPT, Altman DG, Gøtzsche PC, Jüni P, Moher D, Oxman AD (2011). The Cochrane Collaboration’s tool for assessing risk of bias in randomised trials. BMJ (Clinical research ed).

[59] Institute of Medicine of the National Academies (2011). Finding what works in health care: standards for systematic reviews.

[60] Daniel WW (1999). Biostatistics: a foundation for analysis in the health sciences.

[61] Sheu S, Lin HS, Hwang SL (2002). Perceived stress and physio-psycho-social status of nursing students during their initial period of clinical practice: the effect of coping behaviors. Int J Nurs Stud.

[62] Baqutayan S (2015). Stress and coping mechanisms: a historical overview. Mediterr J Soc Sci.

[63] Labrague LJ, McEnroe-Petitte DM, Gloe D, Thomas L, Papathanasiou IV, Tsaras K (2017). A literature review on stress and coping strategies in nursing students. J Ment Health.

[64] Alzayyat A, Al-Gamal E (2014). A review of the literature regarding stress among nursing students during their clinical education. Int Nurs Rev.

[65] Bhurtun HD, Azimirad M, Saaranen T, Turunen H (2019). Stress and coping among nursing students during clinical training: an integrative review. J Nurs Educ.

[66] McCarthy B, Trace A, O’Donovan M, Brady-Nevin C, Murphy M, O'Shea M, O'Regan P (2018). Nursing and midwifery students’ stress and coping during their undergraduate education programmes: an integrative review. Nurse Educ Today.

[67] Labrague LJ, McEnroe–Petitte DM, De Los Santos JAA, Edet OB (2018). Examining stress perceptions and coping strategies among Saudi nursing students: a systematic review. Nurse Educ Today.

[68] Younas A (2016). Levels of stress and coping strategies used by nursing students in Asian countries: an integrated literature review. J Middle East North Afr Sci.

[69] Labrague LJ, McEnroe-Petitte DM, Al Amri M, Fronda DC, Obeidat AA (2018). An integrative review on coping skills in nursing students: implications for policymaking. Int Nurs Rev.

[70] Amr A, El-Gilany A-H, El-Moafee H, Salama L, Jimenez C (2011). Stress among Mansoura (Egypt) baccalaureate nursing students. Pan Afr Med J.

[71] Madian A, Abdelaziz M, Ahmed H (2019). Level of stress and coping strategies among nursing students at Damanhour University, Egypt. Am J Nurs Res.

[72] Abd El All NH, Abou Shousha AAEF. Stress factors and coping strategies as perceived by nursing students = عوامل الضغوط و إستراتیجیات المواجهة من وجهة نظر طلبة كلیة التمریض . Zagazig Nurs J. 2015;11(1):16–32.

[73] Elsayes H, Obied H (2017). Association between senior nursing students’ perceived stress and learning environment in clinical practice. J Nurs Educ Pract.

[74] Khater W, Akhu-Zaheya L, Shaban I (2014). Sources of stress and coping behaviours in clinical practice among baccalaureate nursing students. Int J Humanit Soc Sci.

[75] Alsaqri SH (2017). Stressors and coping strategies of the Saudi nursing students in the clinical training: a cross-sectional study. Educ Res Int.

[76] Aziz F (2012). Stressors and coping strategies among baccalaureate nursing students at Shifa College of Nursing Islamabad, Pakistan. Int J Nurs Educ.

[77] Eswi AS, Radi S, Youssri H (2013). Stress/ stressors as perceived by baccalaureate Saudi nursing students. Middle-East J Sci Res.

[78] Al-Barrak MY, El-Nady MT, Fayad EA (2011). Sources of stress as perceived by nursing students at King Saud University. Med J Cairo Univ.

[79] Watson R, Rehman S, Ali PA (2017). Stressors affecting nursing students in Pakistan. Int Nurs Rev.

[80] Kareem MS (2019). Stress level among nursing students in hawler medical university at Erbil CIty-Iraq. Malays J Nurs.

[81] Basal A (2018). Foundations of stressors and coping behaviors in different clinical practice areas among undergraduate nursing students in faculty of nursing Tanta University.

[82] Mohamed BM, Ahmed ES (2012). Perception of nursing students towards clinical stressors in the Faculty of Applied Medical Sciences – Al Jouf University – Saudi Arabia. J Am Sci.

[83] Aedh AI, Elfaki NK, Mohamed IA (2015). Factors associated with stress among nursing students (Najran University-Saudi Arabia). IOSR J Nurs Health Sci.

[84] El Rafaey NM (2020). Clinical setting related stressors perceived by medical surgical nursing students. J Nurs Health Sci.

[85] Shdaifat E (2018). Stress and coping strategies among nursing students. Global J Health Sci.

[86] Parveen A, Inayat S (2017). Evaluation of factors of stress among nursing students. Adv Pract Nurs.

[87] Afzal M, Hussain M, Waqas A, Sehar N. Sources of stress among the nursing students of private universities of Pakistan. S Am J Nurs. 2016. 10.21522/TIJNR.2015.02.01.Art004.

[88] Bashir R (2014). Sources of stress among nursing students of university of Lahore (LSN): Thesis.

[89] Al Zu’bi MAKM, Shawabka GAF (2018). Assessment of stress factors for nursing student toward training in the critical areas of Princess Basma Hospital. J Middle East North Afr Sci.

[90] Fashafsheh I, Ayed A, Alkaissi A (2015). Stressors affecting baccalaureate nursing students in the clinical area in Palestinian Universities. J Health Med Nurs.

[91] Al-Gamal E, Alhosain A, Alsunaye K (2018). Stress and coping strategies among Saudi nursing students during clinical education. Perspect Psychiatr Care.

[92] Ahmed WAM, Mohammed BMA (2019). Nursing students’ stress and coping strategies during clinical training in KSA. J Taibah Univ Med Sci.

[93] Mohammed Q, Sajit K (2016). Stress and its associated factors among students of the College of Nursing University of Baghdad. Iraqi Natl J Nurs Spec.

[94] Ajweh AM, Shukri R, Kazem A, Saloom W. Stressors and coping strategies among nursing students at the College Nursing in Sultan Qaboos University. Int J Learn Man Sys. 2015;3:51–69.

[95] Hamaideh SH, Al-Omari H, Al-Modallal H (2017). Nursing students’ perceived stress and coping behaviors in clinical training in Saudi Arabia. J Ment Health.

[96] Ismaile S (2017). Open J Nurs.

[97] Subhi Al-Zayyat A, Al-Gamal E (2014). Perceived stress and coping strategies among Jordanian nursing students during clinical practice in psychiatric/mental health courses. Int J Ment Health Nurs.

[98] Shaban IA, Khater WA, Akhu-Zaheya LM (2012). Undergraduate nursing students’ stress sources and coping behaviours during their initial period of clinical training: a Jordanian perspective. Nurse Educ Pract.

[99] Maged R, El A, Hosny H, Ata A (2019). Conflict management styles, assertiveness and stress among nursing students.

[100] Alzayyat A, Al-Gamal E (2016). Correlates of stress and coping among jordanian nursing students during clinical practice in psychiatric/mental health course. Stress Health.

[101] John B, Al-Sawad M (2015). Perceived stress in clinical areas and emotional intelligence among baccalaureate nursing students. J Indian Acad Appl Psychol.

[102] Naqvi A, Alrasheed F, Ahmad R, Ahmad N. Academic stress and prevalence of stress-related self-medication among undergraduate female students of health and non-health cluster colleges of a public sector University in Dammam, Saudi Arabia. J Pharm Bioallied Sci. 2018;9:251–8. 10.4103/jpbs.JPBS_189_17 PMC5810075 29456376

[103] Alghamdi S, Aljabri S, Jafari G, Alzebali R, Alkunaidiri N, Kalantan N (2019). Sources of stress among undergraduate nursing students. Global J Health Sci.

[104] Shalaby S, AlDilh SMS (2015). Exploring the relationship between perceived stress and academic achievement among critical care nursing students. Athens J Health.

[105] Shalaby S, Aljezani A (2018). Exploring the relationship between perceived educational environment and academic achievement among critical care nursing students. Clin Nurs Stud.

[106] Yehia DBM, Jacoub SM, Eser SM (2016). Predictors of coping strategies among nursing college students at AL-Zaytoonah University of Jordan. J Educ Pract.

[107] Khalil Abdulqawi MS, Brair SL. Coping strategies regarding stress among bachelor nursing students in clinical practice, Khartoum State, 2018. Al Neelain Med J. 2018;(22). ISSN 1858-6155.

[108] Hassanein S, Elshair I, Abdrbo A, Hassan E (2016). Coping strategies as predictors of coursework stress among university nursing students. J Nurs Educ Pract.

[109] Al Masaed AS. Relationships of positive and negative psychological stress coping strategies with locus of control and some variables among a sample of Al-al Bayt University students. 2013;7(3):19. مجلة الدراسات التربویة والنفسیة.

[110] Mahfouz R, Alsahli H (2016). Perceived stress and coping strategies among newly nurse students in clinical practice. J Educ Pract.

[111] al Zamil L (2017). Perceived level of stress, & coping strategies among Saudi nursing student. IOSR J Nurs Health Sci.

[112] Ibrahim SR, Sayed FS (2018). Stress level and coping behaviors of psychiatric nursing students pre and post clinical practice. J Nurs Health Sci.

[113] COPE Inventory. Measurement instrument database for the social science. Carver, C.S; 2013. Retrieved from www.midss.ie.

[114] Carver CS (1997). You want to measure coping but your protocol’s too long: consider the brief COPE. Int J Behav Med.

[115] Robotham D, Julian C (2006). Stress and the higher education student: a critical review of the literature. J Furth High Educ.

[116] Folkman S, Lazarus RS (1988). The relationship between coping and emotion: implications for theory and research. Soc Sci Med.

[117] Raymond J, Sheppard K (2017). Effects of peer mentoring on nursing students’ perceived stress, sense of belonging, self-efficacy and loneliness. J Nurs Educ Pract.

[118] Klainin-Yobas P, Keawkerd O, Pumpuang W, Thunyadee C, Thanoi W, He H-G (2014). The mediating effects of coping on the stress and health relationships among nursing students: a structural equation modelling approach. J Adv Nurs.

[119] Seyedfatemi N, Tafreshi M, Hagani H (2007). Experienced stressors and coping strategies among Iranian nursing students. BMC Nurs.

[120] Zhao FF, Lei XL, He W, Gu YH, Li DW (2015). The study of perceived stress, coping strategy and self-efficacy of C hinese undergraduate nursing students in clinical practice. Int J Nurs Pract.

[121] Del Prato D, Bankert E, Grust P, Joseph J (2011). Transforming nursing education: a review of stressors and strategies that support students’ professional socialization. Adv Med Educ Pract.

[122] Ghodasara SL, Davidson MA, Reich MS, Savoie CV, Rodgers SM (2011). Assessing student mental health at the Vanderbilt University School of Medicine. Acad Med.

[123] Ibrahim AK, Kelly SJ, Adams CE, Glazebrook C (2013). A systematic review of studies of depression prevalence in university students. J Psychiatr Res.

[124] Puthran R, Zhang MWB, Tam WW, Ho RC (2016). Prevalence of depression amongst medical students: a meta-analysis. Med Educ.

[125] Nelwati N, McKenna L, Plummer V (2013). Indonesian student nurses’ perceptions of stress in clinical learning: a phenomenological study. J Nurs Educ Pract.

[126] Zupiria Gorostidi X, Huitzi Egilegor X, Jose Alberdi Erice M, Jose Uranga Iturriotz M, Eizmendi Garate I, Barandiaran Lasa M (2007). Stress sources in nursing practice. Evolution during nursing training. Nurse Educ Today.

[127] Lo R (2002). A longitudinal study of perceived level of stress, coping and self-esteem of undergraduate nursing students: an Australian case study. J Adv Nurs.

[128] Maalouf FT, Alamiri B, Atweh S, Becker AE, Cheour M, Darwish H, Ghandour LA, Ghuloum S, Hamze M, Karam E, Khoury B, Khoury SJ, Mokdad A, Meho LI, Okasha T, Reed GM, Sbaity E, Zeinoun P, Akl EA (2019). Mental health research in the Arab region: challenges and call for action. Lancet Psychiatry.

[129] Pulido-Martos M, Augusto-Landa JM, Lopez-Zafra E (2012). Sources of stress in nursing students: a systematic review of quantitative studies. Int Nurs Rev.

[130] Chan CK, So WK, Fong DY (2009). Hong Kong baccalaureate nursing students’ stress and their coping strategies in clinical practice. J Prof Nurs.

[131] Yamashita K, Saito M, Takao T (2012). Stress and coping styles in Japanese nursing students. Int J Nurs Pract.

[132] Penn TD (2008). Enhancing student comfort in psychiatric clinical settings. Teach Learn Nurs.

[133] Zhao FF, Lei XL, He W, Gu YH, Li DW (2015). The study of perceived stress, coping strategy and self-efficacy of Chinese undergraduate nursing students in clinical practice. Int J Nurs Pract.

[134] Yan ATC (2019). Prediction of perceived stress of Hong Kong nursing students with coping behaviors over clinical practicum: a cross-sectional study. J Biosci Med.

[135] Devkota R, Shrestha S (2018). Stress among bachelor level nursing student. Nepal Med Coll J.

[136] Carter A, Elzubeir M, Abdulrazzaq Y, Revel A, Townsend A (2003). Health and lifestyle needs assessment of medical students in the United Arab Emirates. Med Teach.

[137] El-Gilany A-H, Amr M, Hammad S (2008). Perceived stress among male medical students in Egypt and Saudi Arabia: effect of sociodemographic factors. Ann Saudi Med.

[138] Saipanish R (2003). Stress among medical students in a Thai medical school. Med Teach.

[139] Perceived Stress Scale (PSS). From: Cohen S, Kamarck T, Mermelstein R. A global measure of oerceived stress. J Health Soc Behav. 1983;24(4):385–96. 6668417

[140] Jones MC, Johnston DW (1999). The derivation of a brief Student Nurse Stress Index. Work Stress.

[141] Lindop E (1991). Individual stress among nurses in training: why some leave while others stay. Nurse Educ Today.

[142] Ab Latif R, Mat Nor MZ (2019). Stressors and coping strategies during clinical practice among diploma nursing students. Malays J Med Sci.

[143] Turner K, McCarthy VL (2017). Stress and anxiety among nursing students: a review of intervention strategies in literature between 2009 and 2015. Nurse Educ Pract.

[144] Algaralleh A, Altwalbeh D, Alzayyat A (2019). Preliminary psychometric properties of the Arabic version of Sheu and colleagues Perceived Stress Scale among nursing students at Jordanian universities. J Multidiscip Healthc.

[145] Watson R, Deary IJ, Thompson DR, Li G (2010). The stress in nursing students scale (SINS): principal components analysis of longitudinal data from Hong Kong. J Clin Nurs.

[146] Folkman S. Stress: appraisal and coping. In: Gellman MD, Turner JR (eds). Encyclopedia of behavioral medicine. New York; Springer; 2013. 10.1007/978-1-4419-1005-9_215.

