# Supplementary material for: Perceived stress, stressors, and coping strategies among nursing students in the Middle East and North Africa: an overview of systematic reviews
Source: Syst Rev. 2021 May 5;10:136. doi: 10.1186/s13643-021-01691-9 (PMC8101235; doi:10.1186/s13643-021-01691-9)
Supplement: Supplementary file 1 — Additional file 1: Table S1. The 2009 PRISMA checklist for reporting a systematic review. Table S2. PRIO-harms checklist for reporting an overview of systematic reviews (OoSRs). Table S3. Characteristics of the included systematic reviews. Table S4. Prevalence of stress, stressors and coping strategies among nursing students in the MENA countries with available data. Table S5. Quality assessment of included systematic reviews. Table S6. Quality assessment of included studies. [file 13643_2021_1691_MOESM1_ESM.docx]

**Perceived stress, stressors and coping strategies among nursing students in the Middle East and North Africa: An overview of systematic reviews**

AUTHORS

Sonia Chaabane^1^, Karima Chaabna^1^, Sapna Bhagat^1^, Amit Abraham^1^, Sathyanarayanan Doraiswamy^1^, Ravinder Mamtani^1^, Sohaila Cheema^1*^

AFFILIATION

^1^Institute for Population Health, Weill Cornell Medicine - Qatar

CORRESPONDING AUTHOR:

Dr. Sohaila Cheema

Email: [soc2005@qatar-med.cornell.edu](mailto:soc2005@qatar-med.cornell.edu)

**Table S1.** The 2009 PRISMA checklist for reporting a systematic review.

| **Section/topic** | **#** | **Checklist item** | **Reported on page #** |
| --- | --- | --- | --- |
| **TITLE** | | |  |
| Title | 1 | Identify the report as a systematic review, meta-analysis, or both. | 1 |
| **ABSTRACT** | | |  |
| Structured summary | 2 | Provide a structured summary including, as applicable: background; objectives; data sources; study eligibility criteria, participants, and interventions; study appraisal and synthesis methods; results; limitations; conclusions and implications of key findings; systematic review registration number. | 2 |
| **INTRODUCTION** | | |  |
| Rationale | 3 | Describe the rationale for the review in the context of what is already known. | 4 |
| Objectives | 4 | Provide an explicit statement of questions being addressed with reference to participants, interventions, comparisons, outcomes, and study design (PICOS). | 4 |
| **METHODS** | | |  |
| Protocol and registration | 5 | Indicate if a review protocol exists, if and where it can be accessed (e.g., Web address), and, if available, provide registration information including registration number. | 6 |
| Eligibility criteria | 6 | Specify study characteristics (e.g., PICOS, length of follow-up) and report characteristics (e.g., years considered, language, publication status) used as criteria for eligibility, giving rationale. | 7 |
| Information sources | 7 | Describe all information sources (e.g., databases with dates of coverage, contact with study authors to identify additional studies) in the search and date last searched. | 6 |
| Search | 8 | Present full electronic search strategy for at least one database, including any limits used, such that it could be repeated. | 6 |
| Study selection | 9 | State the process for selecting studies (i.e., screening, eligibility, included in systematic review, and, if applicable, included in the meta-analysis). | 7 |
| Data collection process | 10 | Describe method of data extraction from reports (e.g., piloted forms, independently, in duplicate) and any processes for obtaining and confirming data from investigators. | 6 |
| Data items | 11 | List and define all variables for which data were sought (e.g., PICOS, funding sources) and any assumptions and simplifications made. | 8 |
| Risk of bias in individual studies | 12 | Describe methods used for assessing risk of bias of individual studies (including specification of whether this was done at the study or outcome level), and how this information is to be used in any data synthesis. | 8  Supp 35-36 |
| Summary measures | 13 | State the principal summary measures (e.g., risk ratio, difference in means). | N/A |
| Synthesis of results | 14 | Describe the methods of handling data and combining results of studies, if done, including measures of consistency (e.g., I^2^) for each meta-analysis. | N/A |
| Section/topic | # | Checklist item | Reported on page # |
| Risk of bias across studies | 15 | Specify any assessment of risk of bias that may affect the cumulative evidence (e.g., publication bias, selective reporting within studies). | 8 |
| Additional analyses | 16 | Describe methods of additional analyses (e.g., sensitivity or subgroup analyses, meta-regression), if done, indicating which were pre-specified. | 8 |
| **RESULTS** | | |  |
| Study selection | 17 | Give numbers of studies screened, assessed for eligibility, and included in the review, with reasons for exclusions at each stage, ideally with a flow diagram. | 10-13 |
| Study characteristics | 18 | For each study, present characteristics for which data were extracted (e.g., study size, PICOS, follow-up period) and provide the citations. | 10-13  Supp p 11-33 |
| Risk of bias within studies | 19 | Present data on risk of bias of each study and, if available, any outcome level assessment (see item 12). | 10-13  Supp p 42  Supp p 34-35 |
| Results of individual studies | 20 | For all outcomes considered (benefits or harms), present, for each study: (a) simple summary data for each intervention group (b) effect estimates and confidence intervals, ideally with a forest plot. | 10-13  Supp p 11-33 |
| Synthesis of results | 21 | Present results of each meta-analysis done, including confidence intervals and measures of consistency. | 10-13 |
| Risk of bias across studies | 22 | Present results of any assessment of risk of bias across studies (see Item 15). | 10-13  Supp p 34-36 |
| Additional analysis | 23 | Give results of additional analyses, if done (e.g., sensitivity or subgroup analyses, meta-regression [see Item 16]). | N/A |
| **DISCUSSION** | | |  |
| Summary of evidence | 24 | Summarize the main findings including the strength of evidence for each main outcome; consider their relevance to key groups (e.g., healthcare providers, users, and policy makers). | 14-18 |
| Limitations | 25 | Discuss limitations at study and outcome level (e.g., risk of bias), and at review-level (e.g., incomplete retrieval of identified research, reporting bias). | 14-18 |
| Conclusions | 26 | Provide a general interpretation of the results in the context of other evidence, and implications for future research. | 14-18 |
| **FUNDING** | | |  |
| Funding | 27 | Describe sources of funding for the systematic review and other support (e.g., supply of data); role of funders for the systematic review. | 19 |

Source: Moher D, et al 2009 ^1^

Available at: <http://www.prisma-statement.org/documents/PRISMA%202009%20checklist.pdf>

**Table S2**. PRIO-harms checklist for reporting an overview of systematic reviews (OoSRs).

| Section/topic | (Sub-)item# | Checklist item | Reported on page# |
| --- | --- | --- | --- |
| Title  1. Title | 1a | Specify the study design with terms such as ‘‘overview of (systematic) reviews,’’ ‘‘umbrella review,’’ ‘‘(systematic) review of systematic reviews,’’ or ‘‘(systematic) meta-review’’ in the title of the OoSRs. | 1 |
|  | 1b | Mention ‘‘safety’’ or harms related terms, or the adverse event(s) of interest in the title of the OoSRs. | N/A |
| Abstract |  |  |  |
| 2. Structured-like summary | 2a | Provide a structured-like abstract, as applicable: background, objective, data sources, selection criteria, data extraction, review appraisal, data synthesis methods, results, limitations, conclusions. | 2 |
|  | 2b | Report the main findings of analysis of harms undertaken in the OoSRs or/and in the included SRs. | 2 |
| Introduction |  |  |  |
| 3. Rationale | 3a | Specify the rationale and the scope (wide or narrow agendas) for the overview in the context of an existing body of knowledge on the topic. | 4-5 |
|  | 3b | Provide a balanced presentation of potential benefits and harms of the intervention(s). | 4-5 |
|  | 3ca | Define which events are considered harms according to previous literature and provide a clear rationale for the specific harms included in the OoSRs. | 4-5 |
| 4. Objectives (PICOS) | 4 | Provide an explicit statement of research question(s) that specifies PICOS: *Participants *Interventions *Comparators *Outcomes *Study design | 4-5 |
| Methods |  |  |  |
| 5. Protocol and registration | 5a | Indicate if a protocol exists or not. | 6 |
|  | 5b | If registered, provide the name of the registry (such as a valid Web address, PROSPERO). | 6 |
| 6. Eligibility criteria and outcomes of interest | 6a | Specify inclusion and exclusion criteria for study design, participants, interventions, and comparators in detail. | 6-7 |
|  | 6b | List (and define whenever it is necessary) the outcomes for which data were recorded, ideally include prioritization of main and additional outcomes. | 6-7 |
|  | 6c | Include adverse events as (primary or secondary) outcome of interest. Define them and grade their severity (such as mild, moderate, severe, fatal; severity could also be described in the appendix), if appropriate. | 6-7 |
|  | 6db | Specify report characteristics (such as language restrictions, publication status, and years considered) used as criteria for eligibility for the OoSRs (see also item 7). | 6-7 |
| 7. Information sources | 7a | Search at least two electronic databases. | 6 |
|  | 7b | Search supplementary sources (e.g., hand searching, reference lists, related reviews and guidelines, protocol registries, conference abstracts, and other gray literature). | 6 |
|  | 7c | Report the date of last search and/or dates of coverage for each database. | 6 |
| 8. Search strategyc | 8a | Specify full electronic search strategy (algorithm) for at least one database including any limits used (e.g., language and date restrictions-see also subitems 6d and 7c) such that it could be repeated. | 6  Supp 9-10 |
|  | 8b | Present any additional search process (e.g., algorithm or filter for adverse events, searches in pertinent websites) specifically to identify adverse events that have been investigated. | 6 |
| 9. Data management and selection process | 9ad | Describe the software that was used to manage records and data throughout the OoSRs. | 6-9 |
|  | 9b | Define what is an SR and provide the process for selecting SRs and its relevant details (screening the title and abstract or full text by at least two reviewers, selection by multiple independent investigators and resolving disagreements by consensus). | 6-9 |
|  | 9c | Report any attempt to handle overlapping (include one review among multiple potential candidates by choosing for example the most updated SR, the most methodologically rigorous SR or the SR with larger number of primary studies). | 6-9 |
| 10. Additional search for primary studies | 10 | Report additional search to identify eligible primary studies (e.g., searching in more databases or update the search) and its relevant details. | 6 |
| 11. Data collection process | 11a | Describe the method of data extraction from included SRs (e.g., data collection form, extraction in duplicate and independently, resolving disagreements by consensus). | 6-9 |
|  | 11b | Report any processes for obtaining, confirming, or updating data from investigators (e.g., contact with authors of included reviews, obtain data from primary studies of included reviews). | 6-9 |
| 12. Data items | 12 | List (and define whenever is necessary) the variables for which data were recorded (e.g., PICOS items, number of included studies and participants, dose, length of follow up, results, funding sources) and any data assumptions and simplifications made. | 6-9 |
| 13. Assessment of methodological quality and quality of evidence | 13a | State the evaluation of reporting or/and methodological quality (e.g., using PRISMA or PRISMA-harms, AMSTAR or R-AMSTAR) of the included reviews. | 8 Supp 3-7 |
|  | 13be | State the evaluation of quality for individual studies that are included in the SRs (inform whether tools such as Jadad or RoB of Cochrane were used by the included reviews) and for the additional primary studies. | 8 Supp 11-33 |
|  | 13c | State the evaluation of quality of evidence (e.g., using GRADE approach). | 6-9 |
|  | 13d | Describe the methods (e.g., piloted forms, independently, in duplicate) used for the quality assessment. | 8 Supp 34-36 |
| 14. Meta-bias(es) | 14 | Specify any planned assessment of meta-bias(es) (such as publication bias or selective reporting across studies, ROBIS tool). | 5-7 Supp 34-36 |
| 15. Data synthesis | 15a | Specify clearly the method (narrative, meta-analysis, or network meta- analysis) of handling or synthesizing data and their details (e.g., state the principal summary measures that were extracted or calculated, how heterogeneity was assessed, what statistical approaches were used if a quantitative synthesis has been conducted) | 8-9 |
|  | 15b | Describe the software that was used to analyze the data if a quantitative synthesis has been conducted. | N/A |
|  | 15c | Report if zero events are included in the studies and how they were handled in statistical analyses, if relevant. | N/A |
|  | 15d | Describe methods of any prespecified additional analyses (such as sensitivity or subgroup analyses, meta-regression). | 8-9 |
| Results |  |  |  |
| 16. Review and primary study selection | 16a | Provide the details of review selection (e.g., numbers of reviews screened, retrieved, and included and excluded in the overview) and the number of the additional eligible primary studies that were included, ideally with a flow diagram of the overview process. | 10-13 |
|  | 16b | Present a flow diagram that gives separately the number of studies focused on harms outcomes. | 10-13 |
|  | 16cc | List the studies (full citation) that were excluded after reading the full text and provide reasons. | Supp 11-33 |
| 17. Review and primary study characteristics | 17ac | Describe characteristics of each included SR in tables (such as title or author, search date, PICOS, design and number of studies included, number and age range of participants, dose/frequency, follow up period [treatment duration], review limitations, results or conclusion) and of each additional primary study. | 10-13  Supp11-33 |
|  | 17b | For each included SR report language and publication status restrictions that have been used. | 10-13 |
| 18. Overlapping | 18 | Present or/and discuss about overlapping of studies within SRs (at least one of the following):   - Present measures of overlap (such as CCA). - Provide citation matrix.c - Give the number of index publications or/and discuss about overlapping. f | N/A |
| 19. Present assessment of methodological quality and quality of evidence | 19 | Present results in text or/and tablesc of any quality assessment (see also subitems 13a-c):   - Reporting or/and methodological quality of the included SRs. - Inform for the quality of the individual studies that were included in the SRs (report results for sequence generation, allocation concealment, blinding, withdrawals, bias etc.) and for the additional included primary studies. - Quality of evidence. | 10-13  Supp 34-36 |
| 20. Present meta-bias(es) | 20 | Present results of any assessment of meta-bias(es) (such as publication bias or selective reporting across studies, ROBIS assessment). | N/A |
| 21. Synthesis of results | 21a | Summarize and present the main findings of the overview for benefits and harms. If a quantitative synthesis has been conducted, present each summary measure with a confidence interval, prediction interval, or a credible interval and measures of heterogeneity or inconsistency. | 10-13 |
|  | 21b | Give results of any additional analyses, if done (such as sensitivity, subgroup analyses, or meta-regression). | 10-13 |
|  | 21c | Report results for adverse events separately for each intervention. | N/A |
| Discussion |  |  |  |
| 22. Summary of evidence | 22 | Provide a concise summary of the main findings with the strengths and shortcomings of evidence for each main outcome. | 14-18 |
| 23. Limitations | 23a | Discuss limitations of either the overview or included studies (or both) (e.g., different eligibility criteria, limitations of searching reviews, language restrictions, publication and selection bias). | 14-18 |
|  | 23b | Report possible limitations of the included reviews related to harms (issues of missing data and information, definitions of harms, rare adverse effects). | 14-18 |
| 24. Conclusions | 24a | Provide a general interpretation of the results in coherence with the review findings and present implications for practice; consider the harms equally as carefully as the benefits and in the context of other evidence. | 18 |
|  | 24b | Present implications for future research. | 18 |
| Authorship |  |  |  |
| 25. Contributions of authors | 25 | Provide contributions of authors. | 19 |
| 26. Dual (co-)authorship | 26 | Report about dual (co-)authorship in the limitation or declarations of interest section. | N/A |
| Funding |  |  |  |
| 27. Funding or other support | 27a | Indicate sources of financial and other support for the OoSRs (direct funding) or for the authors (indirect funding) or report no funding. | 19 |
|  | 27b | Provide name for the overview funder and/or sponsor, or for the authors’ supporters. | 19 |
|  | 27c | Describe roles of funder(s), sponsor(s), and/or institution(s), if any, in conducted the OoSRs. | 19 |

**Source**: Konstantinos I. et al 2017 ^2^

**Abbreviations**: PRIO-harms: Preferred Reporting Items for OoSRs; SRs: systematic reviews; PICOS: Participants, Interventions, Comparisons, Outcomes, and Study design; CCA: Corrected Covered Area.

^a^ Applicable mainly for OoSRs that focus on adverse events. The description could be placed in methods section.

^b^ Language restrictions, publication status, and years could also be reported in information sources topic-see item 7.

^c^ It could also be placed in an appendix as a supplementary material.

^d^ The software used for the management of the records and data could be placed in the data collection process-see item 11.

^e^ The way of evaluation (e.g., instruments) can be reported in item 19.

^f^ Index publication is the first occurrence of a primary publication in the included reviews. Discussion for overlapping might be placed in the discussion section Search strategies:

**Panel 1: SEARCH STRATEGY IN THE MAIN DATABASES**

**Pubmed**

**Date: 2008- June 21, 2020**

**Filters:** Meta-Analysis; Review; Systematic Reviews, No language restriction

Search ((("Qatar"[Mesh] OR "Bahrain"[Mesh] OR "Oman"[Mesh] OR "Saudi Arabia"[Mesh] OR "Kuwait"[Mesh] OR "United Arab Emirates"[Mesh] OR "Yemen"[Mesh] OR "Egypt"[Mesh] OR "Jordan"[Mesh] OR "Lebanon"[Mesh] OR "Syria"[Mesh] OR "Iraq"[Mesh] OR "Algeria"[Mesh] OR "Libya"[Mesh] OR "Morocco"[Mesh] OR "Tunisia"[Mesh] OR "Djibouti"[Mesh] OR "Sudan"[Mesh] OR "South Sudan"[Mesh] OR "Pakistan"[Mesh] OR "Africa, Northern"[Mesh] OR "Africa, Eastern"[Mesh] OR "middle east"[Mesh] OR "Arabs"[Mesh] OR "UAE"[Text Word] OR "U.A.E"[Text Word] OR Emirat*[Text Word] OR "United Arab Emirates"[Text Word] OR Qatar*[Text Word] OR Oman*[Text Word] OR (Saudi[Text Word] AND Arabia*[Text Word]) OR Saudi*[Text Word] OR Kuwait*[Text Word] OR Bahrain*[Text Word] OR Yemen*[Text Word] OR Egypt*[Text Word] OR Jordan*[Text Word] OR Leban*[Text Word] OR Syria*[Text Word] OR Iraq*[Text Word] OR "West Bank"[Text Word] OR Gaza*[Text Word] OR Palestin*[Text Word] OR Algeria*[Text Word] OR Libya*[Text Word] OR Morocc*[Text Word] OR Tunis*[Text Word] OR Djibouti*[Text Word] OR Sudan*[Text Word] OR South Sudan*[Text Word] OR Pakistan*[Text Word] OR (North[Text Word] AND Africa*[Text Word]) OR North-Africa*[Text Word] OR ("Africa"[Text Word] AND "Northern"[Text Word]) OR "Northern Africa"[Text Word] OR "East Africa"[Text Word] OR ("Africa"[Text Word] AND "Eastern"[Text Word]) OR "Maghreb"[Text Word] OR "Maghrib"[Text Word] OR Arab*[Text Word] OR Bedouin*[Text Word] OR "Gulf Cooperation Council"[Text Word] OR "GCC"[Text Word] OR "Middle East"[Text Word]))) AND ("Stress Disorders, Traumatic"[Mesh] OR "Stress, Psychological"[Mesh] OR "Adaptation, Psychological"[Mesh] OR Coping[Text Word] OR "Psychologic Adaptation"[Text Word] OR "Psychologic Adaptations"[Text Word] OR "Psychological Adaptations"[Text Word] OR "Psychological Adaptation"[Text Word] OR "Adaptive Behavior"[Text Word] OR "Adaptive Behaviors"[Text Word] OR Stress*[Text Word] OR "Mental Suffering"[Text Word] OR Anguish[Text Word] OR "Compassion Fatigue"[Text Word]) Filters: Meta-Analysis; Review; Systematic Reviews; Publication date from 2008/01/01; Humans

**Embase**

**Date: 2008- June 21, 2020**

**Filters:** Human, Meta-Analysis; Systematic Reviews, evidence-based medicine, exclude medline journals, No language restriction

(exp stress/ or Stress.mp. Or Coping.mp. or exp coping behavior/ or Psychologic Adaptation?.mp. Or Psychological Adaptation?.mp. or Adaptive Behavior?.mp. or Stress*.mp. Or Mental Suffering.mp. Or Mental Stress.mp. Or Anguish.mp. Or Compassion Fatigue.mp. Or alarm reaction.mp. Or mental tension.mp. or psychic tension.mp.) AND (exp Middle East/ or exp North Africa/ or exp Arab/ or exp Djibouti/ or exp Pakistan/ or exp Sudan/ or exp South Sudan/ or Middle East.mp. or North Africa.mp. or EMRO.mp. or Eastern Mediterranean.mp. or Arab.mp. or Arabs.mp. or Arab World.mp. or Algeria*.mp. or Bahrain*.mp. or Djibouti.mp. or Egypt*.mp. or Jordan*.mp. or Kuwait*.mp. or Leban*.mp. or Libya*.mp. or Iraq*.mp. or Morocc*.mp. or Oman*.mp. or Pakistan*.mp. or Qatar*.mp. or Saudi*.mp. or Sudan*.mp. or Syria*.mp. or Tunisia*.mp. or United Arab Emirates.mp. or Emirat*.mp. or West Bank.mp. or Ghaza*.mp. or Gaza*.mp. or Palestin*.mp. or Yemen*.mp. or UAE.mp. or KSA.mp.)

limit to ((human and exclude medline journals and (evidence-based medicine or meta-analysis or "systematic review") and yr="2008 -Current"))

**PsycInfo**

**Date: 2008- June 21, 2020**

**Filters:** Meta-Analysis; Meta-synthesis; literature review; Systematic Reviews, No language restriction

(DE "Arabs" OR MA qatar OR bahrain OR oman OR "Saudi arabia" OR saudi OR Kuwait OR Arabia* OR "united arab emirates" OR yemen OR egypt OR jordan OR lebanon OR syria OR Iraq OR algeria OR libya OR morocco OR tunisia OR djibouti OR sudan OR "South sudan" OR pakistan OR "North africa" OR "northern africa" OR "east africa" OR "eastern africa" OR bedouin OR maghreb OR maghrib OR "gulf cooperation council" OR GCC OR SU qatar OR bahrain OR oman OR "Saudi arabia" OR saudi OR Kuwait OR Arabia* OR "united arab emirates" OR yemen OR egypt OR jordan OR lebanon OR syria OR Iraq OR algeria OR libya OR morocco OR tunisia OR djibouti OR sudan OR "South sudan" OR pakistan OR "North africa" OR "northern africa" OR "east africa" OR "eastern africa" OR bedouin OR maghreb OR maghrib OR "gulf cooperation council" OR GCC) OR (TX UAE OR TX U.A.E OR TX Emirat* OR TX United Arab Emirates OR TX Qatar* OR TX Oman* OR TX Saudi Arabia* OR TX Saudi* OR TX Kuwait* OR TX Bahrain* OR TX Yemen* OR TX Egypt* OR TX Jordan* OR TX Leban* OR TX Syria* OR TX Iraq* OR TX West Bank OR TX Gaza* OR TX Palestin* OR TX Algeria* OR TX Libya* OR TX Morocc* OR TX Tunis* OR TX Djibouti* OR TX Sudan* OR TX South Sudan* OR TX Pakistan* OR TX North Africa* OR TX North-Africa* OR TX "Northern Africa" OR RX "East Africa" OR TX "Eastern Africa" OR TX Maghreb OR TX Maghrib OR TX Arab* OR TX Bedouin* OR TX "Gulf Cooperation Council" OR TX GCC OR TX "Middle East") AND (DE "Stress" OR DE "Academic Stress" OR DE "Caregiver Burden" OR DE "Chronic Stress" OR DE "Environmental Stress" OR DE "Financial Strain" OR DE "Minority Stress" OR DE "Occupational Stress" OR DE "Physiological Stress" OR DE "Post-Traumatic Stress" OR DE "Psychological Stress" OR DE "Social Stress" OR DE "Stress Reactions") OR (DE "Stress and Coping Measures")) OR (DE "Stress and Trauma Related Disorders" OR DE "Acute Stress Disorder" OR DE "Adjustment Disorders" OR DE "Attachment Disorders" OR DE "Posttraumatic Stress Disorder")) OR (DE "Stress Management")) OR (DE "Stress Reactions" OR DE "Compassion Fatigue")) OR (DE "Coping Behavior")) OR (DE "Suffering") OR TX ( Stress* OR "Caregiver burden" OR "adjustment disorder" OR "adjustment disorders" OR adversity OR coping OR "compassion fatigue" OR "emotional control" OR helpless* OR resilien* OR "Psychologic Adaptation" OR "Adaptive behavior" OR "Adaptive behaviors" OR "Adaptive behaviour" OR "Adaptive behaviours" OR "Psychologic Adaptation" OR "Psychologic Adaptations" OR "Psychological Adaptation" OR "Psychological Adaptations" OR suffer*))

**Table S3. Characteristics of the included systematic reviews.**

| Systematic Review Citation | Health Outcome | Geographical Coverage | Literature Search Period for primary studies | Data Sources | MENA Countries with Identified Data | Number of Included Studies on MENA |
| --- | --- | --- | --- | --- | --- | --- |
| Labrague et al., 2018 ^3^ | Stress perceptions and coping strategies | Saudi Arabia | 2010-August 2017 | SCOPUS, CINAHL, PubMed, Ovid Manual searches of references of relevant articles | Saudi Arabia (+Oman, Pakistan, Jordan)* | 14 |
| Labrague et al., 2017^4^ | Stress level and coping strategies | Global | 2000-2015 | CINAHL, MEDLINE, PsycINFO, PubMed Manual search of nursing research journals | Jordan | 3 |
| Labrague et al., 2018 a ^5^ | Coping skills for stress | Global | 2002- 2016 | PsycINFO, PubMed, CINAHL, MEDLINE, SCOPUS A manual search of nursing research journals Further search of references on relevant papers | Jordan Egypt Saudi Arabia | 5 |
| Alzayyat et al., 2014^6^ | Stress | Global | 2002-2013 | MEDLINE, CINAHL, PsycINFO, PubMed  The university library catalogue  A manual search through selected journals that were not available electronically | Jordan | 1 |
| Younas, 2016^7^ | Stress and coping strategies | Asian countries | 2007-2014 | PubMed, EMBASE, Cochrane, CINHAL, ASSIA, PsycINFO  Science Direct, Google Scholar The literature search was expanded to research gate, websites, reference lists, Higher Education Commission of Pakistan’s Electronic Library. | Pakistan and Jordan | 2 |
| Bhurtun et al., 2019^8^ | Stress and coping strategies | Global | January 2007-August 2018 | CINAHL, MEDLINE, PsycINFO, SCOPUS, ERIC | Saudi Arabia and Jordan | 5 |
| McCarthy et al., 2018^9^ | Stress and coping strategies | Global | 2010-2016 | CINAHL, PsycINFO, PubMed | Jordan | 2 |
| Actual overview | Stress and coping strategies | 20 MENA countries | 2000-2018 | PubMed, MEDLINE, CINAHL, PsycINFO, SCOPUS, ASSIA, Ovid, Science Direct, ERIC, Google Scholar, Research gate, websites, reference lists, Higher Education Commission of Pakistan’s Electronic Library, electronic and manual searches of the nursing research journals and reference lists of relevant articles, The university library catalogue | Saudi Arabia Jordan, Egypt, Oman, Pakistan | 20 |
|  |  |  | Up to November 2019** | GoogleScholar, OpenGrey, E-Marefa, ALMANHAL platform, and the list of relevant included studies and SRs | Saudi Arabia, Jordan, Palestine, Iraq, Egypt, Pakistan, Bahrain | 20 |

* Studies each on Oman, Pakistan and Jordan identified within text of the SR.

** Search period for primary studies and SRs.

**Table S4. Prevalence of stress, stressors and coping strategies among nursing students in the MENA countries with available data.**

| Systematic Review | Included Study | Outcome | Prevalence (%) | Instrument used / Data Source | Country | Study population | Study design | Study period | Age | Gender | | Sample size | Stressors | Coping Strategies |
| --- | --- | --- | --- | --- | --- | --- | --- | --- | --- | --- | --- | --- | --- | --- |
| Labrague et al., 2018^3^ | Al Zamil et al., 2017 ^10^ | Low stress and coping strategies | 5.2 | PSS  Brief COPE | Saudi Arabia | 2nd to 4th year nursing students | CS | 2015-2016 | 95.9%: 19-24 4.1%>24 | M+F | | 97 | N/R | Strategies used:   - 41.2% nursing students do a lot of (religion) as coping strategy - 51.5% utilized acceptance - 48.5% utilized active coping |
|  |  | Moderate stress and coping strategies | 75.3 |  |  |  |  |  |  |  |  |  |  |  |
|  |  | High stress and coping strategies | 19.6 |  |  |  |  |  |  |  |  |  |  |  |
| Labrague et al., 2018^3^/ Bhurtun et al., 2019^8^ | Mahfouz and Alsahli, 2016^11^ | Moderate stress and coping strategies | 5.9 | PSS  ACOPE | Saudi Arabia | 5th level nursing students | CS | 2015-2016 | 20-23 | F | | 51 | N/R | Coping behaviors reported:   - Seeking diversions - Self-reliance - Developing social support - Solving family problems - Ventilating feelings - Engaging in demanding activities |
|  |  | High stress and coping strategies | 94.1 |  |  |  |  |  |  |  |  |  |  |  |
| Labrague et al., 2018^3^ | Mohamed and Ahmed et al., 2012^12^ | Moderate stress perception | 28 | PSS | Saudi Arabia ((Faculty of Applied Medical Science-Nursing Department –Al-Jouf University) | Nursing students in third and fourth year (levels 5, 6 and 8) | CS | . | 22.82±1.51 | M+F   \|  \| 1.46 \| 0.14 \| \| --- \| --- \| --- \| \|  \| 65 (52%) \|  \| | | 50 | Clinical training stressors: high-taking care of patient, lack of professional knowledge and skills, stress from assignments and work load/Low-stress from clinical environment Education stressors: educational workload, external conditions, and lack of enough break time. Critical care (24%) and intensive care (22%) units were found to be the most stressful clinical settings. Level 8 nursing students had significantly higher stress than those in the lower levels. | N/R |
|  |  | High stress perception | 72 |  |  |  |  |  |  |  |  |  |  |  |
| Labrague et al., 2018^3^ | Shalaby and AlDilh, 2015^13^ | Low stress perception | 65 | PSS | Saudi Arabia | Female nursing students in Critical Care Nursing course | CS | 2016-2017 | 22-28 | F | | 60 | Most common source of students' stress was clinical stressor, followed by intrapersonal, and academic. | N/R |
|  |  | Moderate stress perception | 28.3 |  |  |  |  |  |  |  |  |  |  |  |
|  |  | High stress perception | 6.7 |  |  |  |  |  |  |  |  |  |  |  |
| Labrague et al., 2018a^5^ | Yehia et al., 2016^14^ | Low stress | 3.3 | PSS  Brief COPE | Jordan (Al-Zaytoonah University of Jordan) | Nursing college students | CS | . | 22-25 | M: 87 (30.1%)  F: 184 (69.9) | | 271 | N/R | Strategies used:   - Prying or mediating (religion) - Find comfort in my religion or spiritual beliefs - Taking action to try to make the situation better |
|  |  | Moderate stress | 84.5 |  |  |  |  |  |  |  |  |  |  |  |
|  |  | High stress | 12.2 |  |  |  |  |  |  |  |  |  |  |  |
| Hand searched | Amr et al., 2011^15^ | Low Stress | 59.8 | PSS  A self-report questionnaire for assessing physical well-being factors | Egypt (Mansoura College of Nursing) | Nursing students | CS | 2008-2009 | 17-22 18.8±1.2 | F | | 373 | The five most frequently reported stressors were fear of future, self-reported anxiety and depression, increased class workload, accommodation problems and congested classrooms. Significant differences in the sociodemographic data of those with high and low stress level such as family residence (p= 0.016), father´s education (p=0.015), father´s work (p=0.022) and grade of previous year (p= 0.031). The high stress group had a significant trend for living in rural areas, their fathers were less frequently professional, had lower school  education and grade in the previous year | N/R |
|  |  | High Stress | 40.2 |  |  |  |  |  |  |  |  |  |  |  |
| Hand searched | Madian et al., 2019^16^ | Mild stress | 28.5 | Students profile structured self-administered questionnaire  Stress assessment scale questionnaire  COPE Inventory | Egypt | nursing students enrolled in first, second, third and fourth year. | CS | 2018-2019 | 20.7±1.1. 19-23 | M: 62 (15.5%) F: 338 (84.5%) | | 400 | Multivariate analysis logistic regression for stress level and coping strategies among undergraduates’ students, a statistical significant relation was observed that family income affected the stress level by increase in income reduced stress level OR=26.075 followed by gender as females had higher perceptions of stress compared to males OR=11.301 and BMI as higher stress score were detected among obese and overweight than normal students, OR=3.755 | More than half (52%) of the students had fair coping strategies, compared to one quarter(25%) who classified as poor coping strategies. Significant differences observed between students, academic (p<0.001), personal (p0.052), social (p0.002) or transportation (p<0.001) and coping strategies among the undergraduates’ students(p Religious related strategies comes firstly with a mean of 84.7±24.1 followed by acceptance, self-blaming, positive reframing and planning strategy with a mean of 66.1±32.5, 62.8±32.4, 62.1±29.6 and 61.3±29.5 respectively. While venting, denial and behavioral disengagement reported by less than forty percent of the students (39.7±28.6, 38.6±32.7 and 38.4±28.7 respectively). Significant difference between gender and coping strategies among the students with mean coping strategies (%) in males: 59.4±15.3 and females: 50.5±14.7 (p<0.001) there was a significant differences observed between academic level of students during last term and their coping strategies (p:0.001) Gender affecting the coping strategies (OR=12.809) followed by body mass index (4.8 OR=29) and living with family ( OR= 3.794) with a statistical significant relation. |
|  |  | Moderate stress | 48.3 |  |  |  |  |  |  |  |  |  |  |  |
|  |  | Severe stress | 23.2 |  |  |  |  |  |  |  |  |  |  |  |
| Hand searched | Kareem, 2019^17^ | Low stress | 15.5 | questionnaire comprised of two parts  Part I: Socio-demographic characteristic  Part II: PSS | Iraq (College of Nursing Hawler Medical University) | 50 students from each academic year | CS | April 23rd, 2018 and July 21st, 2018 | 21.06±2.58 81-22: 81% | M: 63 (31.5%) F: 137 (68.5%) | | 200 | No significant association between the level of stress and their sociodemographic characteristics, only with respect to gender was significant statistically (p= 0.029). female students had more stress than males. No statistically significant differences between stages of student's study and levels with stress. | N/R |
|  |  | Moderate stress | 65 |  |  |  |  |  |  |  |  |  |  |  |
|  |  | High stress | 13 |  |  |  |  |  |  |  |  |  |  |  |
| Hand searched | Nevine et al., 2015^18^ | Low stress | 15.5 | PSS  Copying strategies tool | Egypt (Faculty of Nursing Damanhour University) | Nursing students in the four academic years 2013-2014 (year I, year II, year III, year IV) | CS | 23/4/2014 to 22/5/2014 | 18-24+ | M: 93 (28.3%) F: 237 (71.7%) | | 330 | Highest percentage of highly stress was from assignment and work load about three-fourth (79.0%) and the second one of highly stress was stress from peers and daily life (72.7%) and the remaining four sub-items of highly stress could be ranked in descending order as stress from nursing clinical teachers and nursing staff (59.0%), stress from taking care of patients (55.3%) , stress from clinical environment (52.3%), and the last one was stress from lack of professional nursing knowledge and skills (44.1%). There were a significant differences in perceived stress level of nursing students and their characteristics in each of the following: the academic year (p = 0.000) the highest percentages of highly stress was in second year (74.6%).,   family monthly income (p= 0.005) the highest percentages of highly stress (73.3%) was in inadequate income.  Hours spent for studying (p=0.016) with highest percentages of stress level (74.4%) when spending 6 hrs studying and hours of sleeping/ night (p=0.001) with highest percentages of stress level (74.4%) when having 6 hrs of sleep. There were significant differences in both of the academic year and the hours of sleeping at night and their physio-psycho- social stress (0.000, 0.004 respectively) The highest percentage of highly stress in both of them was academic year four was (45.8%) and they were sleeping at night for 8 hours and more (46.2%) | Main strategies adopted by nursing students were ranked in descending order as follow:   - Transferences (43.2%) - Staying optimistic (23.4%) - Problem solving (17.0%) - Avoidance (16.4%) |
|  |  | Moderate stress | 60.8 |  |  |  |  |  |  |  |  |  |  |  |
|  |  | High stress | 23.7 |  |  |  |  |  |  |  |  |  |  |  |
|  |  | Low stress | 13.7 | Physio-psycho-social stress scale  Copying strategies tool |  |  |  |  |  |  |  |  |  |  |
|  |  | Moderate stress | 65 |  |  |  |  |  |  |  |  |  |  |  |
|  |  | High stress | 21.3 |  |  |  |  |  |  |  |  |  |  |  |
| Hand searched | Naqvi et al., 2018^19^ | Low Academic stress | 13.6 | PSS  questionnaire | Saudi Arabia (Imam Abdulrahman Bin Faisal University including nursing college) | 22 nursing students among undergraduate female students (n=386)  of different colleges | CS | January 2017- May 2017 | 20.7 | F | | 22 | Predictors of stress: Examination load (85%), Course load (74.1%), Assignments load(66.6%), Lack of free time during semesters (62.5%), cGPA (70.8%), Career and future prospects (52%). Students of preparatory year had low perceived stress, whereas those of 2nd and 4th year mainly had moderate to high perceived stress. Students of 3rdyear had moderate perceived stress, whereas students of 5th and 6th year reported low perceived stress. These findings were statistically significant (p = 0.0001) | N/R |
|  |  | Moderate Academic stress | 63.6 |  |  |  |  |  |  |  |  |  |  |  |
|  |  | High Academic stress | 22.7 |  |  |  |  |  |  |  |  |  |  |  |
| Hand searched | Mohammed et al., 2016^20^‬‬‬‬‬‬‬‬‬‬‬‬‬‬‬‬‬‬‬‬‬‬‬‬‬‬‬‬‬‬‬‬‬‬‬‬‬‬‬‬‬‬‬‬‬‬‬‬‬‬‬‬‬‬‬‬‬‬‬‬‬‬‬‬‬‬‬‬‬‬‬‬‬‬‬‬‬‬‬‬‬‬‬‬‬‬‬‬‬‬‬‬‬‬‬‬‬‬‬‬‬‬‬‬‬‬‬‬‬‬‬‬‬‬‬‬‬‬‬‬‬‬‬‬‬‬‬‬‬‬‬‬‬‬‬‬‬‬‬‬‬‬‬‬‬‬‬‬‬‬‬‬‬‬‬‬‬‬‬‬‬‬‬‬‬‬‬‬‬‬‬‬‬‬‬‬‬‬‬‬‬‬‬‬‬‬‬‬‬‬‬‬‬‬‬‬‬‬‬‬‬‬‬‬‬‬‬‬‬‬‬‬‬‬‬‬‬‬‬‬‬‬‬‬‬‬‬‬‬‬‬‬‬‬‬‬‬‬‬‬ | Low stress | 0.8 | scale of perceived stress with 29 items and cut off point for the total score as low=29 -67.5, moderate= 67.6 -96.5 and high = 96.6- 145 questionnaire contained factors induced stress which categorized as: environmental, intrapersonal, academic and interpersonal | Iraq (College of Nursing/ University of Baghdad) | undergraduate nursing students | CS | November 1st, 2014 to May 1st, 2015 | . | M: 73 (57%) F: 55 (43%) | | 128 | Environmental stressors: 85.9% of them perceive living environmental change, 69.5% of them perceive inadequate safety and security, 72.7% perceived lack of recreational facilities, 64.8% of them perceive the absence of calm environment and 72.7% of them perceive the inadequate of water provision Intrapersonal stressors: the majority of them were perceived feeling of homesick (78.9%), change in sleeping pattern (78.1%), and decline in personal health (70.3%) Academic stressors: all of them (100%) perceive the lack of rest between lecture, 78.1% of them experience the imbalance between holiday and study time, all the student (100%) perceive the burdens of the study and getting lower grade than anticipation, 70.3% of them unable to enjoy the study and 77.3% perceive the stressor of practical training Interpersonal stressors: the high percentages were 47.7% to fight with close one and 51.6% perceive the conflict with roommate | N/R |
|  |  | High stress | 99.2 |  |  |  |  |  |  |  |  |  |  |  |
| Hand searched | Basal et al., 2018^21^ | Low stress | 52 | Sociodemographic characteristics questionnaire PSS CBI | Egypt | Undergraduate nursing students second, third and fourth academic years | CS | 2016-2017 | Range: 19-25 Mean: 20.72±1.072 | M: 104 (26.1%) F: 294 (73.9%) | | 398 | Highest domain of stress was from working with various kinds of patients (39.98±12.745) compared to stress from lack of professional knowledge and skills (6.37±3.152). Other stressors were Stress factors due to the training environment, Stress from the evaluation by supervisors, Stressors due to the career, Stress from teachers and nursing staff, stress from assignments and workload No a statistical significant difference between the three levels of stress and students’ academic year (p=0.073) | Most common coping behavior utilized:   - Problem solving followed by - Staying optimistic Statistically significant difference between the three levels of stress and the score of coping behavior (P =0.018) Highly significant negative correlation between the total score of stress and the four domains of coping behaviors |
|  |  | Moderate stress | 26 |  |  |  |  |  |  |  |  |  |  |  |
|  |  | High stress | 22 |  |  |  |  |  |  |  |  |  |  |  |
| Labrague et al., 2018^3^ | Ajweh et al., 2015 ^22^ | Stress perception | . | Stressor scale with 35 items  Coping strategies scale with 66 items | Oman | Nursing students in 3^rd^ and 4^th^ year during clinical training | . | 2011-2012 | . | M+F | | 109 | Clinical sources of stress include worry over taking care of patients including dying patients and patients with terminal illnesses | Higher utilization of passive coping |
| Labrague et al., 2018^3^ / Labrague et al., 2017^4^/ Bhurtun et al., 2019^8^ | Al Zayyat et al., 2016^23^/ Al-Zayyat et al., 2014 a^23^ | Higher Stress perception | . | PSS  CBI | Jordan | Nursing students Clinical Practice in Psychiatric/Mental Health Course | LS | 2012-2013 | 21.15yrs 20-25 | M: 10 (15.4%)  F: 55 (84.6%) | | 65 | Demographics to be important in explaining stress perceptions.  Students who were in the fourth year, with a low family income, who avoid extracurricular activities, with a low academic grade or who registered in other clinical course(s) reported high stress degrees. | - Problem solving - Staying optimistic   Students who utilized avoidance or transference strategies reported high stress degrees. |
| Labrague et al., 2018^3^ | Watson et al., 2017^24^ | Stress perception | . | Questionnaire Urdu translated version of SINS | Pakistan | High level nursing students from 11 schools of nursing | CS | . | <18-45 | M: 276 (38%) F: 450 (62%) | | 726 | Increasing workloads Greater responsibility Increasing professional stressors as they progressed up the various academic levels, and perhaps increased pressure by their peers from the lower academic levels. The total score on the Stressors in nursing students scale was related to gender with males scoring higher. The score generally increased over 4 years of the program, and students in private schools of nursing scored higher than those in public schools of nursing. | N/R |
| Labrague et al., 2018^3^ | Al Barrak et al., 2011^25^ | Stress perception and coping strategies | . | SSS | Saudi Arabia | Level 1 to level 7  nursing students | CS | . | 18-35 | F | | 298 | Academic stressor: increased workload, anticipation of graduation  Intrapersonal stressor: changes in sleeping habits, eating habits, new responsibilities Interpersonal stressors: change in sleeping habits, change in eating habits, new responsibilities Environmental stressors: college discipline, non-flexible and long working hours, change in living conditions Student's academic level (highest stress was among fifth and sixth-level students, 80.0% and 90.0%, respectively p=0.009) and age (highest among students in the age group 20-21 years 64.3%, p=0.04) correlated significantly with stress. Marital status of student nurses correlated significantly with interpersonal stressors with married students experiencing higher stress levels. | N/R |
| Labrague et al., 2018^3^ | Aedh et al., 2015^26^ | Stress perception and coping strategies | . | PSS | Saudi Arabia | Year 2 to year 5 students | CS | May-Oct2015 | 21.3±1.2 | M+F | | 50 | Sources of stress: lack of professional and skills, assignment and workload, taking care of patient.  Students (higher level) academic year was associated with student's stress. | Students reported higher preference for the following coping styles:   - Transference - Staying optimistic |
| Labrague et al., 2018^3^ | Alsaqri, 2017^27^ | Stress perception and coping strategies | . | PSS; CBI | Saudi Arabia | Junior and senior nursing students | CS | Sept-Dec 2016 | ≤ 21-25+ | M: 85 (42%)  F: 115 (58%) | | 200 | Top sources of stress: assignments and workload, lack of professional knowledge and skills, and from clinical environment.  Gender and interest in nursing were not significantly related to stress perceptions.  Students in the age group 22–24 years had significantly more stress. | Coping behaviors most commonly utilized were:   - Problem solving followed by - Staying optimistic - Transference |
| Labrague et al., 2018^3^/ Bhurtun et al., 2019^8^ | Al-Gamal et al., 2017^28^ | Stress perception | . | PSS  CBI | Saudi Arabia | Nursing students  in clinical practice | CS | 2015-2016 | 21.4 20-24 | F | | 121 | Higher stress originated from taking care of the patient (x=12.0, ̅SD = 5.00), from the teachers and nursing staff (x=10.2, SD = 3.55), and from the assignments and workload (x=8.9, SD = 3.00). | The most frequently used coping strategies:   - Problem-solving strategy followed by - Avoidance coping strategy.   The total PSS score and the problem-solving coping strategy subscale score (r=−0.352, p=0.0005) has low negative significant relationship |
| Labrague et al.,2018^3^ | Eswi et al., 2013^29^ | Stress perception and coping strategies | . | PSS  ICSRLE | Saudi Arabia | Nursing students | CS | . | 22.5 | F | | 100 | Sources of stress: lot of responsibilities, grades than hoped for (43.0%), not enough sleep (43.0%), too many things required at the same time (42.0%), no enough leisure time (39.0%), important decisions about future career (38.0%), long waits to get service (32.0%).  Perceived stress and students recent life experiences (p < 0.01; r=0.47) significantly correlated.  Perceived stress has no correlation with recent students' life experiences, age, and marital status, number of dependents, academic level and stream. | N/R |
| Labrague et al., 2018^3^/ Labrague et al., 2018a^5^ | Hamaideh et al., 2017^30^ | Stress perception | . | PSS  CBI | Saudi Arabia | 2nd to 4th year students | CS | . | 22.48 20-27 | M: 45 (45%)  F: 55 (55%) | | 100 | Sources of stress: assignments and workload, from teachers and nursing staff, from the environment. | Common coping behaviors utilized:   - Problem-solving - Staying optimistic - Transference. |
| Labrague et al., 2018^3^ | Ismaile, 2017^31^ | Stress and coping strategies | . | PSS | Saudi Arabia | Nursing students | CS | 2015-2016 | 22.65 21-25 | F | | 55 | For level 7 students, sources of stress were from: teachers and nursing staff, taking care of patients, assignments and workloads. For level 8 students, sources of stress were from: taking care of patients (x=14.55, SD = 3.36), teachers and nursing staff (x=13.82 SD = 4.06), assignments and workloads (x=12.16, SD = 4.62). | N/R |
| Labrague et al., 2017^4^/ Labrague et al., 2018a^5^/ Bhurtun et al., 2019^8^/ McCarthy et al., 2018^9^ | Al-Zayyat et al., 2014 c ^32^ | Stress and coping strategies | . | PSS  CBI | Jordan | Nursing students during pre- and post-clinical periods in PMHN courses. | LS | 2012-2013 | 21.15 20-25 | M: 10 (15.4%)  F: 55 (84.6%) | | 65 | Type of clinical stressors:  Caring for patients, assignments and workloads, teachers and staff nurses | - Problem-solving strategy - Staying optimistic strategy followed by - Avoidance - Transference |
| Labrague et al., 2017^4^/ Labrague et al., 2018a^5^/ Alzayyat et al., 2014^6^  / Bhurtun et al., 2019^8^/ McCarthy et al., 2018^9^ | Shaban et al., 2012^33^ | Coping strategies related to their perceived university course work stress | . | PSS  CBI | Jordan | 2^nd^ year Nursing students in initial clinical practice | CS | September and November 2010. | 19.8yrs 19-21y | F:131 (72.4%)  M: 50 (27.6%) | | 181 | Type of clinical stressors:  Assignment, clinical environment, staff nurses and teachers, stress from caring for patients, worry about grades, lack of professional skills and knowledge, pressure from the nature and quality of clinical practice | - Problem solving - Staying optimistic - Transference - Avoidance   Inadequate knowledge, skills, peers and daily life which correlated negatively with optimistic coping strategies |
| Labrague et al., 2018 a^5^ | Hassanein et al., 2016^34^ | Stressors and coping strategies | . | RWCSQ | Egypt | Nursing students | CS | 2011-2012 | 20.5±1.27 | F:48 (50%)  M: 48 (50%) | | 96 | N/R | - Problem-focused coping - Seeking social - Support - Detachment - Wishful thinking - Focusing on the positive - Tension reduction |
| Younas, 2016^7^ | Aziz et al., 2012^35^ | Stressors and coping strategies | . | SSCI | Pakistan (Shifa college of Nursing, Islamabad) | baccalaureate nursing students of all years | Analytical CS | . |  |  | | 78 | Stressors: nursing theory, clinical experience, college environment and social/personal environment.  No association among demographic variables and levels of stress. | Most common coping strategies used:   - Discuss feeling with friends or classmates - Did what is expected of me - Self-analysis to understand the situation better) - Accept the situation - Become involved in other activities. |
| Younas, 2016^7^ | Khater et al., 2014^36^ | Stressors and coping strategies | . | Self-reported questionnaire, which is composed of demographic data  PSS  CBI | Jordan | Nursing students in their second, third and fourth year | CS | September 2010 and November 2010. | 20.9 (±1.4) 19-29 | M: 208 (34.8%) F: 389 (65.2%) | | 597 | The most common type of stressors perceived was stress from assignment work (M= 2.12, SD=0.88), followed by stress of peers and daily life (M=1.65, SD= 0.91) and from nursing staff and teachers (M =1.58, SD=0.89)  Significant negative low correlation between student’s **age** and stress (r=-.14, p< .05)  There were significant mean differences between **students’ interest** (whether students possess an interest or not in nursing) and students’ perception of stress (t =2.38, p< .05). student’s **academic year** was directly associated with the student's stress level (F = 8.34; df = 2, 594, p<.05) students in the second year had significantly more stress (M= 1.70) than students in the third (M= 1.51) and fourth (M= 1.45) academic year **Courses students enrolled in** were found to be closely related to their stress levels. (F = 7.75; df = 5, 591, p <.05).Students enrolled in Adult Health Nursing I (M= 1.77) course had significantly more stress than students enrolled in Adult Health Nursing II (M= 1.42), Pediatric (M= 1.66), Maternal Child Health (M= 1.51), Critical (M= 1.33), and Training course (M= 1.52) | The most common coping behavior utilized:   - Problem solving (M= 2.4; SD= 0.87), followed by - Staying optimistic (M= 2.3; SD = 0.8) - Transference (M= 2.11; SD=0.99) - Avoidance was less frequently utilized (M= 1.4; SD= 0.76) |
| Hand searched | Ahmed et al., 2019^37^ | Stress and coping strategies | . | Questionnaire of demographic variables  PSS  CBI | Saudi Arabia | Nursing students enrolled in clinical training at any academic level | CS | . | 21(±1.56) | M: 60 (48%) F: 65 (52%) | | 125 | Main external influencing stressors: noise (20; 16%), moving location (nine; 7.2%), social interaction (seven; 5.6%), and personal illness (seven; 5.6%). Stress related to  providing care was 1.56(0.45) assignments and capacity was 1.60(0.43)  deficiency of professional knowledge and competences was 1.24(0.45)  environment of practice was 1.47(0.49)  peers and daily life events was 1.89(0.67)  instructors and nursing staff was 1.45(0.79) | Coping strategies utilized were:   - Taking a problem-solving approach (1.84 ±0.67) - Staying optimistic (1.56 ±0.76) - Transference (1.34 ±1.20) - Avoidance behavior (1.23 ±056) **Regression relationship between stressors and coping strategies used by students: Avoidance:** significant relationship with stressors related to - Assignments & patient care (0.12) - Peers & daily life (0.15), - Teachers & nursing staff (0.16) - Not significantly related to stressors related to - Patient care (0.03) - Lack of professional knowledge & skills (0.02) - Environment (0.03) **Problem solving:**  significant relationship with stressors related to - Assignments & patient care (0.21), - Environment (0.27), Teachers & nursing staff (0.21) and - Not significantly related to stressors related to - Patient care (0.04) - Lack of professional knowledge & skills (0.016) - Peers & daily life (0.04) **Staying optimistic**: significant relationship with - Patient care (0.16) - Assignments & patient care (0.26) - Environment (0.23) and - Not significantly related to - Lack of professional knowledge & skills (0.03) - Peers & daily life (0.07) - Teachers & nursing staff (0.03) **Transference:** significant relationship with Stressors related to - Patient care (0.31) - Assignments & patient care (0.28) - Environment (0.19), and - Not significantly related to Stressors related to - Teachers & nursing staff (0.04) - Lack of professional knowledge & skills (0.06) - Peers & daily life (0.05) |
| Hand searched | Afzal et al., 2016^38^ | Stressors | . | The questionnaire of stressors among nursing students (Academic sources, clinical sources, personal and environmental sources) | Pakistan | nursing students of private institutes enrolled for Post RN BS Nursing, 2 years and BS Nursing 4-year degree program | CS | January2016 - May 2016 | 18-50  <20: 4.9%  20-25: 49.2% 26-30: 39.3% >30: 6.6% | | M: 48 (39.3%) F: 74 (60.7%) | 122 | Academic Sources of Stress 1 Frequent graded activities are very stressful 2 Stress level increases due to getting lower grades than anticipation. 3 Challenging courses and missing too many classes contribute to stress. 4 Inability to balance study and leisure time is source of stress. 5 Inconsiderate and insensitive instructors also cause stress. 6 Lack of expected career advancement, promotion and fear of future create stress. Clinical Sources of Stress 1 Maintaining a balance between clinical work and studying increases stress. 2 New clinical situations, unfamiliar patient’s diagnosis and treatment promote stress level. 3 Differences between the ideal practices learned in school and real situations in the healthcare environment cause of stress. 4 Humiliating behavior of physicians and being criticized in front of patients is reason of stress. 5 Unfriendliness from more senior staff and fear of making mistakes in patient care is a cause of stress in clinical setting. Personal Sources as Stressors 1 Changes in sleeping pattern/lack of sleep enhance stress. 2 Financial problems are main source of stress. 3 Decline in personal health contribute a lot to stress 4 Lack of confidence and inability to decision making enhance stress. Environmental Sources as Stressors 1 Lack of recreational facilities/activities during semester is a source of stress. 2 Difficulties with transportation also a source of stress. 3 Not enough leisure time creates frustration 4 Absence of calm and quite environment in class increases stress level. 5 Congested class room creates anxiety  Nursing student’s responded **highest factor of stress to the academic sources** of stress as the mean value of 4.2842 show that majority of the students were agree and strongly agree on the point that academic sources create stress. Similarly, the nursing students consider clinical sources, personal sources and environmental sources as stressors respectively. However, environmental sources are considered as the lowest source of stress than the other 3 categories. | N/R |
| Hand searched | Parveen et al., 2017^39^ | Stressors | . | Questionnaire divided into four sections: Section A cover the demographic data of the participants. Section B questions related to academic factors of stress. Section C questions consist of clinical factors of stress. Section D questions related to environmental factors of stress. | Pakistan (Allied Hospital, Faisalabad) | Nursing students of diploma in general | CS | . | 16-25  <20: 48.7% 20-25: 51.3% | F | | 150 | Academic sources of stress: getting lower grades than anticipation, Inability to balance study and leisure time is source of stress, lack of expected career advancement, fear of future create stress Clinical factors of stress**:** maintaining a balance between clinical work and studying increases stress, humiliating behavior of physicians and being criticizes in front of patient is reason of stress, fear of making mistake in clinical placement, criticism from peers and senior staffs, inadequate and poor equipment’s, inadequate information about patients from doctors. Environmental factors of stress**:** Lack or recreational facilities during semester is a source or factor of stress, security risk in area of job. | N/R |
| Hand searched | Ibrahim and Sayed, 2018^40^ | Stressors and coping strategies | . | questionnaire was divided into three parts: Part I: socio-demographic characteristic  Part II: PSS, Part III: CBI | Egypt (Faculty of Nursing, Benha University) | nursing students enrolled in 4th academic year undergoing the psychiatric mental health nursing training | A quasi - experimental (pre and post-test) design | 2015-2016 | 22.05±0.70 | M: 18 (18.0%) F: 82 (82.0%) | | 100 | minimal improvement in stress level among Psychiatric Nursing students regarding taking care of patients, stress from assignment and workload and lack of professional knowledge and skills pre-clinical practice were 27.0 ,41.0 and 11.0% compared by post-clinical practice were 10.0, 38.0 and 7.0% respectively. minimal improvement regarding stress from clinical environment, from peers &daily life and from teachers and nursing staff pre-clinical practice were 34.0, 25.0 & 25.0 compared by post clinical practice were 23.0, 18.0 & 20.0 respectively. | Psychiatric Nursing Student more frequently used avoidance and problem solving in pre-clinical practice were 40.0 & 38.0 compared by post clinical practice were 49.0& 41.0 respectively. More frequently used coping strategies regarding optimistic and transference coping behavior in pre-clinical practice were 31.0 & 26.0 compared by post clinical practice were 34.& 40.0 respectively. Highly statistically significant correlations between stress level and coping behaviors of the study sample pre and post clinical practice(p 0.000) |
| Hand searched | Fashafsheh et al., 2015^41^ | Stressors | . | A structured questionnaire included biographical and SCSS | Palestine (6 governmental and private hospitals in Palestinian Universities) | baccalaureate nursing students in clinical training | CS | March 2012 to August 2012 | 18- >25 20-22y: 77% | M: 199 (50.9%) F: 192 (49.1%) | | 396 | Stress factors due to the career: response degree was medium (2.52), while the items of (Smelling or unfavorable odors in the hospital surrounding (3.10) and Being subject for hazards while performing (3.05) had the high response degree among the stressors due to the career  Stress factors due to the training environment**:** response degree was medium (2.59). While the items of Discrepancy between learned procedures and those seen to be practiced at the hospital (3.14) and Training in noisy place (3.18) had the high response degree among the stress factors due to the training environment. Working with various kinds of patients : response degree was medium (2.81). While the items of Training with patient in life threatening situations (3.33), Dealing with mentally disturbed patient (3.08), Handling human excretion (3.44), Being responsible for big numbers of patients (2.71), and Dealing with patient or his family(3.25 ) had the high response degree among the Working with various kinds of patients the Evaluation by supervisors: response degree was medium (2.59) Significant differences between Stress factors due to the career, Stress factors due to the training environment, Working with various kinds of patients, and The evaluation by supervisors (0.001, 0.002, 0.018, and 0.019) respectively and universities at (α =0.05) level. At the same time, there were significant differences between the total domains and the universities (0.002) at the level (α =0.05). Significant differences between the students' levels of study. The results show that the more the level, the higher the degree of stress  there are no significant differences between males and females, marital status, place of residence, and age of the groups | N/R |
| Hand searched | Alghamdi et al., 2019^42^ | Stressors | . | SNSI sociodemographic characteristics questionnaire | Saudi Arabia (King Abdulaziz University, the faculty of Nursing) | Female second-year nursing students | CS | . | 18-24 95.4%: 19-21 | F | | 87 | Academic load: Major source of stress. The highest mean for the items included under academic load was the amount of classwork material to be learned, and examination and/or grades (M = 3.77, SD = 1.24)   Interface worries: The second source of stress reported by the students was (M = 3.22, SD = 0.79). The highest mean for the items included under interface worries was a lack of free time (M = 3.76, SD = 1.35).  The third source of stress was Clinical concerns (M = 2.80, SD = 0.78), and The fourth source of stress was Personal problems (M = 2.43, SD = 1.02). Significant relationships between academic load and monthly income (R = -.320, P ≤ 0.05), and personal problems and monthly income (R = -.360, P ≤ 0.05). However, there were no significant relationships between clinical concerns, interface worries, and monthly income. | N/R |
| Hand searched | Al Zu'bi et al., 2018^43^ | Stressors | . | Interviewing questionnaire with 2 parts: 1. sociodemographic data 2. factors of stress related to (a) workload (b) lack of support and  involvement (c) patient and  family (d) student satisfaction of being a critical area nurses | Jordan (Jordan University of Science and Technology) | Saudi adult females, enrolled in the faculty of nursing  in the  second, third, and fourth-year academic levels. | CS | November 2015- January 2016 | 18-25 | | F | 103 | Workload: shortage of staff and lack of time to spend with the patients were the most stressing factors for the training students Lack of support and involvement: lack of support from college was the most stressing factor as reported by the study participants (2.64±0.39), followed by the lack of support from instructors (2.18±0.51).The least reported were lack of friendly working conditions (1.06±0.55)  and having skills adequate for the critical areas (1.09±0.53)  Patient and family: “patients’ families making unreasonable demand” was the highly estimated stress factors (2.18±0.67) followed by “being blamed for anything that goes wrong” that got a 2.01±0.56 score. | N/R |
| Hand searched | Bashir, 2014^44^ | Stressors | . | A self-structured questionnaire that is composed of 20 close ended questions in 4 categories: academic, clinical, personal and environmental | Pakistan (nursing department of university of Lahore) | Nursing students enrolled in BS(RN) and BScN degree program | CS | March 2014 to May 2014 | 20 to 50  20-25: 54.8% 26-30: 33.9% | M: 28 (45.2%) F: 34 (54.8%) | | 62 | Academic stress: 87.1% students reported stress due to study related concerns(Frequent graded activities, getting lower grades then anticipation, Challenging courses and missing too many classes contribute to stress, Inability to balance study and leisure time , Inconsiderate and insensitive instructors, Lack of expected career advancement, promotion and fear of future),  Clinical sources: reported by 77.4% (Maintaining a balance between clinical work and studying, New clinical situations, unfamiliar patient’s diagnosis and treatment, Differences between the ideal practices learned in school and real situations in the healthcare environment, Humiliating behavior of physicians and being criticized in front of patients, Unfriendliness from more senior staff and fear of making mistakes in patient care) as a source of stress  Personal sources: reported by75.8% (Changes in sleeping pattern/lack of sleep, Financial problems, decline in personal health, Lack of confidence and inability to decision making)  Environmental sources: reported by 71.6% (Lack of recreational facilities/ activities during semester, Difficulties with transportation, Not enough leisure time, Absence of calm and quite environment in class, Congested class room creates anxiety) | N/R |
| Hand searched | El Rafaey et al, 2020^45^ | Stressors | . | Stressors perceived by medical surgical nursing students at clinical setting assessment questionnaire | Egypt Matrouh general hospital”medical and surgical unites” | Medical surgical nursing students performing their clinical training  firstly, contacted with patient in medical surgical clinical setting | descriptive correlational study | . | . | Male  38  (43.2%)  female  50  (56.8%) | | 88 | 1. Perceived stressors related to dealing with patient and relatives: 2. Being in emergency situation was experienced as extremely stressful (22.7%), 3. Patient acuity (18.2%), 4. Sex and age of patient (11.4%), 5. Patient’s physical status (9.1%), 6. Relationship with patients' family members (6.8%) 7. Meeting the patient psychomotor requirements (2.3%)   Perceived stressors related to dealing with nursing staff and new hospital environment,   1. Exposure to contagious diseases (34.1%) 2. Hospital physical environment (22.7%)   Perceived stressors related to the ability to perform the clinical responsibilities,   1. Insufficient time to do things (20.5%), 2. Own abilities in clinical practice (18.2%), 3. fatigue and energy level (15.9%) 4. Possibility to make errors (11.4%)   Perceived stressors related to being evaluated and continuously supervised,   1. Being student” not trusted from patients and their families” (45.5%) 2. Being evaluated from instructors and their ability to answer the question of patients and their families (34.1%)   There was no statistically significant difference between students perceived stressors and their area of residence (P=0.490), sex (P=0.079), religious (P= 0.183)  There was no statistically significant correlation between the student level of perceives stressors and their sociodemographic characteristics as age, sex, religious, place of residence, and GPA of the last term (p= .331, .79, .183, .490, .397) respectively |  |
| Hand searched | Khalil et al., 2018^46^ | Coping strategies | . | CBI | Sudan, seventeen nursing colleges in Khartoum | Bachelor nursing students during their first exposure to clinical practice in medical surgical courses | CS | . | <20  (59.00%)  ≥20  (41.00%) | Male  52 (11.7%)  394 Female (88.3%) | | 446 | . | The most common coping behavior method utilized in dealing with stressful clinical events was:   1. To keep an optimistic and positive attitude in dealing with life events (3.14 ± 1.21) 2. Having confidence in overcoming that difficulties (3.02 ± 1.15) 3. Seeing things objectively (2.90 ± 1.21) 4. Setting up objectives to solve problems (2.86 ± 1.25) 5. Having confidence in performing as well as senior schoolmates (2.85 ± 1.22)   The least common coping behaviors used were:   1. To quarrel with others and lose temper (1.00 ± 1.34) 2. To avoid teachers (1.26 ± 1.34) 3. To expect miracles to avoid facing difficulties (1.13 ± 1.34) |
| Hand searched | Masaed et al., 2012^47^ | Coping strategies | . | instrument for coping  instrument for locus of control | Jordan (Al al-Bayt University) | Nursing college students | CS analytical | 2010-2011 | . | M: 37 (35.2%) F: 68 (64.8%) | | 105 | N/R | Positive coping strategies utilized:   - Asking others for help - Show their feelings and express their reaction - Use of religion, prayer, invocation - Physical exercise and relaxation - Problem understanding and solving   Nursing students with external locus of control had a score of 21.28 in using positive coping strategies |
| Hand searched | Elsayes et al., 2018^48^ | Perceived stress and coping strategies | . | Nursing Students’ Perception of Stress in Clinical Practice Questionnaire which has 2 parts: 1) Personal data, 2) PSS Physio-Psycho-Social Response Scale Coping Strategies Scale Dundee Ready Education Environment Measure | Egypt (Faculty of nursing-Tanta University) | Senior nursing students in their 4th year during clinical training | CS | N/R | < 21: 130 (32.5%)  21-23: 264 (66%)  >23: 6 (1.5%) | M: 75 (18.8%) F: 325 (81.2%) | | 400 | The highest three stressors in clinical environment as perceived by senior nursing students were regarding assignment & workload, teachers & nursing staff and peer & daily life Moderate level of stress regarding lack of knowledge and skills  High statistically significant correlation between senior nursing students’ perception of learning environment and their responses to stress. Statistical high significant difference between students enrolled in community course and students enrolled in psychiatric nursing and nursing administration courses total responses to stress and total perception of learning environment at p < .01. Also community nursing students experienced higher levels of stress than of psychiatric nursing and nursing administration courses | Majority of participant senior nursing students sometimes and always used (problem and emotional focused disengagement coping strategies):   - Wishful thinking - Self-criticism - Social withdrawal - Problem avoidance   High statistically significant negative correlation between their perception of learning environment and their coping strategies at p < .001 |
| Hand searched | Shdaifat et al., 2018^49^ | Stress and coping strategies | . | PSS  CBI | Saudi Arabia (University of Dammam) | Nursing students | Cross-sectional | November - April 2017 | Mean age: 23.9 (SD=5.07) | M: 124 (67.4%) F: 60 (32.6%) | | 184 | Assignment and workload: Most common (M=1.82, SD=0.90), students felt stressed when they were worry about bad grades, experience pressure of clinical practice, and not meet teachers’ expectations. Teacher and nursing staff: second common (M=1.80, SD=0.83), such as experience discrepancy between theory and practice, do not know how to discuss patients’ illness with teachers, etc Taking care of patients: Third common (M=1.47,SD=0.86) as Lack of experience and ability in providing nursing care, do not know how to help patients with physio-psycho-social problems etc. Environment : least sources of stress (M=1.28, SD=0.90) such as; feel stressed in the hospital environment where clinical practice takes place, unfamiliar with the ward facilities, and feel stressed from the rapid change in patient’s condition A negative week correlation between stress level and income (r= -0.22, p= 0.024) Gender and smoking were statistically significantly different with stress level t (182) = -2.37, p=0.019 and t (180) = -1.89, p=0.027, respectively | Most frequent coping strategy was   - Problem solving (M=2.53, SD=0.87) for example, setting up objectives, adopting strategies to solve problems, making plans and listing priorities, finding the meaning of stressful incidents, and employing experience. - Staying optimistic (M=2.28, SD=0.73) was the second coping strategies - Avoidance was the least coping strategies used (M=1.17, SD=0.61)   Positive moderate correlation between “Avoidance” and all the PSS subscales, with “Stress from taking care of patients” (r= 0.33, p= 0.001), “Stress from teachers and nursing staff” (r= 0.26, p= 0.001), “Stress from assignments and workload” (r= 0.40, p= 0.001), “Stress from peers and daily life” (r= 0.29, p= 0.001), “Stress from lack of professional knowledge and skills” (r= 0.30, p= 0.001), and “Stress from the environment” (r= 0.35, p= 0.001)  “Problem solving” was correlated with “Stress from taking care of patients” (r= -0.16, p= 0.04), and with “Stress from peers and daily life” (r= -0.17, p= 0.03) Gender was statistically significantly with coping behavior using “Problem solving” level t (169) = 2.44, p=0.016 and type residence had statistically significantly with coping behavior using Transference level t (180) = -3.11, p=0.002 |
| Hand searched | Maged et al., 2018^50^ | Mild stress | 10.1 | SNSI | Egypt (Faculty of nursing, Zigazig University) | Nursing students from four academic years | Descriptive correlational | March-April, 2017 | Mean age: 20.56±1.314 | M: 96 (22.6%) F: 328 (77.4%) | | 424 | Highest percentages of mean scores were for academic load (67.6%), followed by interface worries (63.68%), then clinical (practical) concerns (51.42%); while, the lowest percent of mean scores was for personal problems (47.05%) Statistically significant positive correlation between nursing students' total stress score and their using of conflict management compromising style with their clinical instructors (P < 0.05) Negative statistically significant correlation between nursing students' total stress score and their using of conflict management compromising style with peers (P = 0.01) Negative statistically significant correlation between nursing students' total mean scores of assertiveness and stress (P = 0.03). | . |
|  |  | Moderate stress | 43.5 |  |  |  |  |  |  |  |  |  |  |  |
|  |  | High stress ø | 46.4 |  |  |  |  |  |  |  |  |  |  |  |
| Hand searched | John et al., 2015^51^ | Stress | . | PSS | Bahrain (College of Health Sciences, University of Bahrain) | Nursing students from second year to fourth year | Descriptive correlational | May 2012 to February 2013 | 135 | 18-35 | | M: 23 (17%) F: 112 (83%) | Highest mean stress levels in terms of assignments and workload, and was the highest among the fourth year students followed by stress from peers and daily life Incomparable stress level from lack of professional knowledge and skills as denoted by p-value of .006 No significant differences in the perceived stress levels of students in the clinical areas when grouped according to their gender except for 2 indicators: Stress from assignments and workload, with a t-value of -2.667 and p-value of 0.012, at 0.05 level of significance shows that males can handle stress better than their female counterparts Stress from lack of professional knowledge and skills, p-value of .040 shows that males have more stress than females A highly negative significant relationship (r value of -0.276 and p-value of .001) between the over-all stress level and EI (Emotional Intelligence) was observed among the respondents tested at 0.01 level of significance | . |

**PSS:** Perceived Stress Scale, **CBI:** The Coping Behaviors Inventory, **SCSS:** Student Clinical Stressor Scale, **RWCSQ:** Revised Ways of Coping Strategies Questionnaire

**SNSI**: Student Nurse Stress Index**, SSCI:** Students Stress and Coping Inventory, **SSS:** Student Stress survey, **SINS:** The Stressors in Nursing Students scale, **COPE**: Coping Orientation to Problems Experienced, **Brief COPE**: abbreviated version of the full COPE Inventory, **CS:** Cross-sectional, **LS**: Longitudinal study, **M**: Males, **F**: Females, **ICSRLE:** Inventory of College Students’ Recent Life Experiences; **BMI:** body mass index

Ø High stress includes severe and highly severe stress

**Table S5. Quality assessment of included systematic reviews**

| Systematic Review Citation | 1.Was an ' a priori' design provided? | 2.Was there duplicate study selection and data extraction? | 3.Was a comprehensive literature search performed? | 4. Was the status of publication (i.e. grey literature) used as an inclusion criterion? | 5.Was a list of studies (included and excluded) provided? | 6.Were the characteristics of included studies provided? | 7.Was the scientific quality of included studies assessed and documented? | 8. Was the scientific quality of included studies used appropriately in formulating conclusions? | 9.Were the methods used to combine the findings of studies appropriate? | 10.Was the likelihood of publication bias assessed? | 11.Was the conflict of interest included? |
| --- | --- | --- | --- | --- | --- | --- | --- | --- | --- | --- | --- |
| Labrague et al., 2018^3^ | No | No | Yes | No | No | Yes | Yes | Yes | No | N/A | No |
| Labrague et al., 2017 ^4^ | No | No | Yes | No | No | Yes | Yes | Yes | No | N/A | No |
| Labrague et al., 2018 a^5^ | No | No | Yes | No | No | Yes | Yes | Yes | No | N/A | No |
| Alzayyat et al., 2014^6^ | No | No | Yes | Yes | No | Yes | Yes | Yes | No | N/A | No |
| Younas, 2016^7^ | No | No | Yes | Yes | No | Yes | No | No | No | N/A | No |
| Bhurtun et al., 2019^8^ | No | No | No | No | No | Yes | Yes | Yes | No | N/A | No |
| McCarthy et al., 2018^9^ | No | No | No | No | No | Yes | Yes | Yes | No | N/A | No |

Notes: We used the AMSTAR checklist with additional notes made by Michelle Weir, Julia Worswick, and Carolyn Wayne based on conversations with Bev Shea and/or Jeremy Grimshaw in June and October 2008 and July and September 2010. Available on <https://amstar.ca/docs/AMSTARguideline.pdf>

The absence of a statement regarding any criteria of the quality assessment was considered as not done. The unit of analysis for the systematic reviews is the study. The unit of analysis for the actual overview is the systematic review.

Abbreviations: N/A: Not applicable;

† To get a ‘yes’ for the included SRs, the conflict of interest should be clearly acknowledged for the SR and the included original studies.

To get a ‘yes’ for the actual overview, the conflict of interest should be clearly acknowledged for the actual overview and all included SRs.

**Table S6. Quality assessment of included studies**

| Systematic Review citation | Included study citation | 1.Were study participants sampled in an appropriate way? | 2.Was the sample size adequate? | 3a. Were the study subjects described in detail? | 3b. Was the setting described in detail? | 4. Were valid methods used for the identification of the condition? |
| --- | --- | --- | --- | --- | --- | --- |
| Labrague et al., 2018^3^ | **Ajweh et al., 2015 ^22^** | Low risk of bias | Low risk of bias | Low risk of bias | Low risk of bias | High risk of bias |
| Labrague et al., 2018^3^ | **Al Zayyat and Al Gamal, 2016^23^** | Low risk of bias | High risk of bias | Low risk of bias | Low risk of bias | Low risk of bias |
| Labrague et al., 2018^3^ | **Watson et al., 2017^24^** | Low risk of bias | Low risk of bias | Low risk of bias | Low risk of bias | Low risk of bias |
| Labrague et al., 2018^3^ | **Al Barrak et al., 2011^25^** | High risk of bias | Low risk of bias | Low risk of bias | Low risk of bias | Low risk of bias |
| Labrague et al., 2018^3^ | **Al Zamil, 2017^10^** | Low risk of bias | High risk of bias | Low risk of bias | Low risk of bias | Low risk of bias |
| Labrague et al., 2018^3^ | **Aedh et al., 2015^26^** | High risk of bias | High risk of bias | Low risk of bias | Low risk of bias | Low risk of bias |
| Labrague et al., 2018^3^ | **Alsaqri, 2017^27^** | Low risk of bias | Low risk of bias | Low risk of bias | Low risk of bias | Low risk of bias |
| Labrague et al., 2018^3^ | **Al-Gamal et al., 2017^28^** | High risk of bias | Low risk of bias | Low risk of bias | Low risk of bias | Low risk of bias |
| Labrague et al., 2018^3^ | **Eswi et al., 2013^29^** | Low risk of bias | Low risk of bias | Low risk of bias | Low risk of bias | Low risk of bias |
| Labrague et al., 2018^3^ | **Hamaideh et al., 2017^30^** | Low risk of bias | Low risk of bias | Low risk of bias | Low risk of bias | Low risk of bias |
| Labrague et al., 2018^3^ | **Ismaile, 2017^31^** | Low risk of bias | High risk of bias | Low risk of bias | Low risk of bias | Low risk of bias |
| Labrague et al., 2018^3^ | **Mahfouz and Alsahli, 2016^11^** | Unclear | High risk of bias | Low risk of bias | Low risk of bias | Low risk of bias |
| Labrague et al., 2018^3^ | **Mohamed and Ahmed, 2012^12^** | Low risk of bias | High risk of bias | Low risk of bias | Low risk of bias | Low risk of bias |
| Labrague et al., 2018^3^ | **Shalaby and AlDilh, 2015^13^** | High risk of bias | High risk of bias | Low risk of bias | Low risk of bias | Low risk of bias |
| Labrague et al., 2017 ^4^ | **Al-Zayyat & Al-Gamal 2014c ^32^** | Low risk of bias | High risk of bias | Low risk of bias | Low risk of bias | Low risk of bias |
| Labrague et al., 2017 ^4^ | **Shaban, 2012^33^** | High risk of bias | Low risk of bias | Low risk of bias | Low risk of bias | Low risk of bias |
| Labrague et al., 2018 a^5^ | **Hassanein et al., 2016^34^** | High risk of bias | High risk of bias | Low risk of bias | Low risk of bias | Low risk of bias |
| Labrague et al., 2018 a^5^ | **Yehia et al., 2016^14^** | High risk of bias | Low risk of bias | Low risk of bias | Low risk of bias | Low risk of bias |
| Younas, 2016^7^ | **Khater et al., 2014^36^** | High risk of bias | Low risk of bias | Low risk of bias | Low risk of bias | Low risk of bias |
| Younas, 2016^7^ | **Aziz et al., 2012^35^** | Unclear | High risk of bias | Low risk of bias | Low risk of bias | Low risk of bias |
| Hand searched | **Afzal et al., 2016^38^** | High risk of bias | Low risk of bias | Low risk of bias | Low risk of bias | High risk of bias |
| Hand searched | **Parveen et al., 2017^39^** | High risk of bias | Low risk of bias | Low risk of bias | Low risk of bias | High risk of bias |
| Hand searched | **Kareem, 2019^17^** | High risk of bias | Low risk of bias | Low risk of bias | Low risk of bias | Low risk of bias |
| Hand searched | **Ibrahim and Sayed,2018^40^** | Low risk of bias | Low risk of bias | Low risk of bias | Low risk of bias | Low risk of bias |
| Hand searched | **Nevine et al., 2015^18^** | Low risk of bias | Low risk of bias | Low risk of bias | Low risk of bias | Low risk of bias |
| Hand searched | **Fashafsheh et al., 2015^41^** | Unclear | Low risk of bias | Low risk of bias | Low risk of bias | Low risk of bias |
| Hand searched | **Madian et al., 2019^16^** | Low risk of bias | Low risk of bias | Low risk of bias | Low risk of bias | Low risk of bias |
| Hand searched | **Ahmed et al., 2019^37^** | Low risk of bias | Low risk of bias | Low risk of bias | Low risk of bias | Low risk of bias |
| Hand searched | **Naqvi et al., 2018^19^** | High risk of bias | High risk of bias | Low risk of bias | Low risk of bias | Low risk of bias |
| Hand searched | **Amr et al., 201^15^** | Low risk of bias | Low risk of bias | Low risk of bias | Low risk of bias | Low risk of bias |
| Hand searched | **Alghamdi et al., 2019^42^** | High risk of bias | High risk of bias | Low risk of bias | Low risk of bias | Low risk of bias |
| Hand searched | **AlZu'bi et al., 2018^43^** | High risk of bias | Low risk of bias | Low risk of bias | Low risk of bias | High risk of bias |
| Hand searched | **Bashir, 2014^44^** | Low risk of bias | High risk of bias | Low risk of bias | Low risk of bias | High risk of bias |
| Hand searched | **Mohammed et al., 2016^20^‬‬‬‬‬‬‬‬‬‬‬‬‬‬‬‬‬‬‬‬‬‬‬‬‬‬‬‬‬‬‬‬‬‬‬‬‬‬‬‬‬‬‬‬‬‬‬‬‬‬‬‬‬‬‬‬‬‬‬‬‬‬‬‬‬‬‬‬‬‬‬‬‬‬‬‬‬‬‬‬‬‬‬‬‬‬‬‬‬‬‬‬‬‬‬‬‬‬‬‬‬‬‬‬‬‬‬‬‬‬‬‬‬‬‬‬‬‬‬‬‬‬‬‬‬‬‬‬‬‬‬‬‬‬‬‬‬‬‬‬‬‬‬‬‬‬‬‬‬‬‬‬‬‬‬‬‬‬‬‬‬‬‬‬‬‬‬‬‬‬‬‬‬‬‬‬‬‬‬‬‬‬‬‬‬‬‬‬‬‬‬‬‬‬‬‬‬‬‬‬‬‬‬‬‬‬‬‬‬‬‬‬‬‬‬‬‬‬‬‬‬‬‬‬‬‬‬‬‬‬‬‬‬‬‬‬‬‬‬‬** | High risk of bias | Low risk of bias | Low risk of bias | Low risk of bias | High risk of bias |
| Hand searched | **Masaed et al., 2012^47^** | Low risk of bias | Low risk of bias | Low risk of bias | Low risk of bias | High risk of bias |
| Hand searched | **Basal et al., 2018^21^** | Low risk of bias | Low risk of bias | Low risk of bias | Low risk of bias | Low risk of bias |
| Hand searched | **Elsayes et al., 2018^48^** | Unclear | Low risk of bias | Low risk of bias | Low risk of bias | Low risk of bias |
| Hand searched | **Shdaifat et al., 2018^49^** | High risk of bias | Low risk of bias | Low risk of bias | Low risk of bias | Low risk of bias |
| Hand searched | **Maged et al., 2018^50^** | Low risk of bias | Low risk of bias | Low risk of bias | Low risk of bias | Low risk of bias |
| Hand searched | **John et al., 2015^51^** | Low risk of bias | Low risk of bias | Low risk of bias | Low risk of bias | Low risk of bias |
| Hand searched | **El Rafaey et al , 2020^45^** | Unclear | High risk of bias | Low risk of bias | Low risk of bias | Low risk of bias |
| Hand searched | **Khalil et al., 2018^46^** | Unclear | Low risk of bias | Low risk of bias | Low risk of bias | Low risk of bias |
| Number of studies with low risk of bias (A) out of total number of included studies(B): A/B | | **20/42**  **(47.6%)** | **28/42**  **(67.5)** | **42/42**  **(100%)** | **42/42**  **(100%)** | **35/42**  **(82.5%)** |
| Number of studies with unclear item | | **6/42** | **0** | **0** | **0** | **0** |

**Unclear**= not reported; **Sampling method**: Probability sampling= Low risk of bias, Non probability sampling= High risk of bias; **For sample size adequacy:** if >100 there is low risk of bias; if <100 there is high risk of bias, **Study subject description:** we considered the following: 1. specific population 2. gender 3. Age. If all 3 parameters reported= low risk of bias; if only 1 reported= high risk of bias; **Setting:** if reported, low risk of bias; **Valid tools employed for the measurement of outcome:** low risk of bias

**REFERENCES**

1 Moher, D., Liberati, A., Tetzlaff, J. & Altman, D. G. Preferred reporting items for systematic reviews and meta-analyses: the PRISMA statement. *Journal of clinical epidemiology* **62**, 1006-1012, doi:10.1016/j.jclinepi.2009.06.005 (2009).

2 Bougioukas, K. I., Liakos, A., Tsapas, A., Ntzani, E. & Haidich, A.-B. Preferred reporting items for overviews of systematic reviews including harms checklist: a pilot tool to be used for balanced reporting of benefits and harms. *Journal of clinical epidemiology* **93**, 9-24, doi:<https://doi.org/10.1016/j.jclinepi.2017.10.002> (2018).

3 Labrague, L. J., McEnroe–Petitte, D. M., De Los Santos, J. A. A. & Edet, O. B. Examining stress perceptions and coping strategies among Saudi nursing students: A systematic review. *Nurse Education Today* **65**, 192-200, doi:<https://doi.org/10.1016/j.nedt.2018.03.012> (2018).

4 Labrague, L. J. *et al.* A literature review on stress and coping strategies in nursing students. *J Ment Health* **26**, 471-480, doi:10.1080/09638237.2016.1244721 (2017).

5 Labrague, L. J., McEnroe-Petitte, D. M., Al Amri, M., Fronda, D. C. & Obeidat, A. A. An integrative review on coping skills in nursing students: implications for policymaking. *International Nursing Review* **65**, 279-291, doi:10.1111/inr.12393 (2018).

6 Alzayyat, A. & Al‐Gamal, E. A review of the literature regarding stress among nursing students during their clinical education. *International Nursing Review* **61**, 406-415 (2014).

7 Younas, A. Levels of Stress and Coping Strategies Used by Nursing Students in Asian Countries: An Integrated Literature Review. *The Journal of Middle East and North Africa Sciences* **2**, 50-57, doi:10.12816/0032673 (2016).

8 Bhurtun, H. D., Azimirad, M., Saaranen, T. & Turunen, H. Stress and Coping Among Nursing Students During Clinical Training: An Integrative Review. *The Journal of nursing education* **58**, 266-272, doi:10.3928/01484834-20190422-04 (2019).

9 McCarthy, B. *et al.* Nursing and midwifery students' stress and coping during their undergraduate education programmes: An integrative review. *Nurse Education Today* **61**, 197-209, doi:<https://doi.org/10.1016/j.nedt.2017.11.029> (2018).

10 al Zamil, L. Perceived Level of Stress, & Coping Strategies among Saudi Nursing Student. *IOSR Journal of Nursing and Health Science* **06**, 06-13, doi:10.9790/1959-0603060613 (2017).

11 Mahfouz, R. & Alsahli, H. Perceived stress and coping strategies among newly nurse students in clinical practice. *J. Educ. Pract.* **7**, 118-128 (2016).

12 Mohamed, B. M. & Ahmed, E. S. Perception of nursing students towards clinical stressors in the Faculty of Applied Medical Sciences – Al Jouf University – Saudi Arabia. *J Am Sci* **8**, 608-617 (2012).

13 Shalaby, S. & Aljezani, A. Exploring the relationship between perceived educational environment and academic achievement among critical care nursing students. *Clinical Nursing Studies* **7**, 1, doi:10.5430/cns.v7n1p1 (2018).

14 Yehia, D. B. M., Jacoub, S. M. & Eser, S. M. Predictors of Coping Strategies among Nursing College Students at AL-Zaytoonah University of Jordan. *Journal of Education and Practice* **7**, 149-154 (2016).

15 Amr, A., El-Gilany, A.-H., El-Moafee, H., Salama, L. & Jimenez, C. Stress among Mansoura (Egypt) baccalaureate nursing students. *Pan Afr Med J* **8**, 26-26, doi:10.4314/pamj.v8i1.71083 (2011).

16 Madian, A., Abdelaziz, M. & Ahmed, H. Level of Stress and Coping Strategies among Nursing Students at Damanhour University, Egypt. *American Journal of Nursing Research* **7**, 684-696, doi:10.12691/ajnr-7-5-3 (2019).

17 Kareem, M. S. STRESS LEVEL AMONG NURSING STUDENTS IN HAWLER MEDICAL UNIVERSITY AT ERBIL CITY-IRAQ. *The Malaysian Journal of Nursing* **11**, 82-88 (2019).

18 Abd El All, N. H. & Abou Shousha, A. A. E. F. Stress Factors and Coping Strategies as Perceived by Nursing Students = عوامل الضغوط و إستراتيجيات المواجهة من وجهة نظر طلبة كلية التمريض. *Zagazig Nursing Journal* **2015 Vol.11 Issue 1, pp.16-32**, 1-17 (2015).

19 Naqvi, A., Alrasheed, F., Ahmad, R. & Ahmad, N. Academic Stress and Prevalence of Stress-Related Self-Medication among Undergraduate Female Students of Health and Non-Health Cluster Colleges of a Public Sector University in Dammam, Saudi Arabia. *Journal of Pharmacy & Bioallied Sciences* **9**, doi:10.4103/jpbs.JPBS_189_17 (2018).

20 Mohammed, Q. & Sajit, K. Stress and Its Associated Factors among Students of the College of Nursing University of Baghdad. *Iraqi National Journal of Nursing Specialties* **29**, 30-37 (2016).

21 Basal, A. Foundations of Stressors and Coping Behaviors in Different Clinical Practice Areas among Undergraduate Nursing Students in Faculty of Nursing Tanta University. (2018).

22 Aisha, M., Ajweh, Shukri, R., Kazem, A. & Saloom, W. Stressors and Coping Strategies among Nursing Students at the College Nursing in Sultan Qaboos University. **3**, 51-69, doi:10.12785/ijlms/030103 (2015).

23 Alzayyat, A. & Al-Gamal, E. Correlates of Stress and Coping among Jordanian Nursing Students during Clinical Practice in Psychiatric/Mental Health Course. *Stress Health* **32**, 304-312, doi:10.1002/smi.2606 (2016).

24 Watson, R., Rehman, S. & Ali, P. A. Stressors affecting nursing students in Pakistan. *International Nursing Review* **64**, 536-543, doi:10.1111/inr.12392 (2017).

25 Al-Barrak, M. Y., El-Nady, M. T. & Fayad, E. A. Sources of stress as perceived by nursing students at King Saud University. *Med J Cairo Univ* **79**, 541-553 (2011).

26 Aedh, A. I., Elfaki, N. K. & Mohamed, I. A. Factors associated with stress among nursing students (Najran University-Saudi Arabia). *IOSR Journal of Nursing and Health Science (IOSR-JNHS)* **4**, 33-38 (2015).

27 Alsaqri, S. H. Stressors and Coping Strategies of the Saudi Nursing Students in the Clinical Training: A Cross-Sectional Study. *Education Research International* **2017**, 8, doi:10.1155/2017/4018470 (2017).

28 Al-Gamal, E., Alhosain, A. & Alsunaye, K. Stress and coping strategies among Saudi nursing students during clinical education. *Perspectives in Psychiatric Care* **54**, 198-205, doi:10.1111/ppc.12223 (2018).

29 Eswi, A. S., Radi, S. & Youssri, H. Stress/ stressors as perceived by baccalaureate Saudi nursing students. *Middle East Journal of Scientific Research* **14**, 193-202, doi:10.5829/idosi.mejsr.2013.14.2.734 (2013).

30 Hamaideh, S. H., Al-Omari, H. & Al-Modallal, H. Nursing students’ perceived stress and coping behaviors in clinical training in Saudi Arabia. *Journal of Mental Health* **26**, 197-203, doi:10.3109/09638237.2016.1139067 (2017).

31 Ismaile, S. Perceived Clinical Stressors among Saudi Nursing Students. *Open Journal of Nursing* **Vol.07No.04**, 10, doi:10.4236/ojn.2017.74036 (2017).

32 Subhi Al-Zayyat, A. & Al-Gamal, E. Perceived stress and coping strategies among Jordanian nursing students during clinical practice in psychiatric/mental health courses. *Int. J. Ment. Health Nurs.* **23**, 326-335, doi:10.1111/inm.12054 (2014).

33 Shaban, I. A., Khater, W. A. & Akhu-Zaheya, L. M. Undergraduate nursing students’ stress sources and coping behaviours during their initial period of clinical training: A Jordanian perspective. *Nurse Education in Practice* **12**, 204-209, doi:<https://doi.org/10.1016/j.nepr.2012.01.005> (2012).

34 Hassanein, S., Elshair, I., Abdrbo, A. & Hassan, E. Coping strategies as predictors of coursework stress among university nursing students. *Journal of Nursing Education and Practice* **7**, doi:10.5430/jnep.v7n2p99 (2016).

35 Aziz, F. Stressors and Coping Strategies Among Baccalaureate Nursing Students at Shifa College of Nursing Islamabad, Pakistan. *International Journal of Nursing Education* **4**, 193 (2012).

36 Khater, W., Akhu-Zaheya, L. & Shaban, I. Sources of Stress and Coping Behaviours in Clinical Practice among Baccalaureate Nursing Students. *International Journal of Humanities and Social Science* **4**, 194-202 (2014).

37 Ahmed, W. A. M. & Mohammed, B. M. A. Nursing students' stress and coping strategies during clinical training in KSA. *Journal of Taibah University Medical Sciences* **14**, 116-122, doi:<https://doi.org/10.1016/j.jtumed.2019.02.002> (2019).

38 Afzal, M., Hussain, M., Waqas, A. & Sehar, N. Sources of Stress among the Nursing Students of Private Universities of Pakistan. *South American journal of nursing* (2016).

39 A Parveen & S Inayat. Evaluation of factors of stress among Nursing Students. *Advanced Practices in Nursing* **02**, doi:10.4172/2573-0347.1000136 (2017).

40 Ibrahim, S. R. & Sayed, F. S. Stress Level and Coping Behaviors of Psychiatric Nursing Students Pre and Post Clinical Practice. *Journal of Nursing and Health Science* **Volume 7**, PP 54-64 (2018).

41 Fashafsheh, I., Ayed, A. & Alkaissi, A. Stressors Affecting Baccalaureate Nursing Students in the Clinical Area in Palestinian Universities. *Journal of Health, Medicine and Nursing* **14**, 42-52 (2015).

42 Alghamdi, S. *et al.* Sources of Stress Among Undergraduate Nursing Students. *Global Journal of Health Science* **11**, 116, doi:10.5539/gjhs.v11n9p116 (2019).

43 Al Zu’bi, M. A. K. M. & Shawabka, G. A. F. Assessment of Stress Factors for Nursing Student toward Training in the Critical Areas of Princess Basma Hospital. *The Journal of Middle East and North Africa Sciences* **2018 Vol.4 Issue 4, pp.8-12**, 1-5 (2018).

44 Bashir, R. *Sources of stress among nursing students of university of Lahore (LSN)*, University of Lahore, (2014).

45 Noura Mahmoud El Rafaey. Clinical Setting Related Stressors Perceived By Medical Surgical Nursing Students. *Journal of Nursing and Health Science* **9**, 01-05 (2020).

46 Khalil Abdulqawi M. S & Sara Lavinia Brair. Coping Strategies Regarding Stress among Bachelor Nursing Students in Clinical Practice, Khartoum State, 2018. *Al Neelain Medical Journal* (2018).

47 Al Masaed, A. S. Relationships of Positive and Negative Psychological Stress Coping Strategies with Locus of Control and Some Variables among a Sample of Al-al Bayt University Students. *2013* **7**, 19, doi:10.24200/jeps.vol7iss3pp256-274 (2013).

48 Elsayes, H. & Obied, H. Association between senior nursing students’ perceived stress and learning environment in clinical practice. *Journal of Nursing Education and Practice* **8**, 126, doi:10.5430/jnep.v8n3p126 (2017).

49 Shdaifat, E. Stress and Coping Strategies Among Nursing Students. *Global Journal of Health Science* **10**, doi:10.5539/gjhs.v10n5p33 (2018).

50 Maged, R., El, A., Hosny, H. & Ata, A. Conflict Management Styles, Assertiveness and Stress among Nursing Students. 49-59, doi:10.9790/1959-0702084959 (2019).

51 John, B. & Al-Sawad, M. Perceived stress in clinical areas and emotional intelligence among baccalaureate nursing students. *Journal of the Indian Academy of Applied Psychology* **41**, 75-84 (2015).
